# Estimation of peptide elongation times from ribosome profiling spectra

**DOI:** 10.1093/nar/gkab260

**Published:** 2021-04-22

**Authors:** Michael Y Pavlov, Gustaf Ullman, Zoya Ignatova, Måns Ehrenberg

**Affiliations:** Department of Cell and Molecular Biology, Biomedical Center, University of Uppsala, 75237 Uppsala, Sweden; Department of Cell and Molecular Biology, Biomedical Center, University of Uppsala, 75237 Uppsala, Sweden; Institute for Biochemistry & Molecular Biology, University of Hamburg, 20146 Hamburg, Germany; Department of Cell and Molecular Biology, Biomedical Center, University of Uppsala, 75237 Uppsala, Sweden

## Abstract

Ribosome profiling spectra bear rich information on translation control and dynamics. Yet, due to technical biases in library generation, extracting quantitative measures of discrete translation events has remained elusive. Using maximum likelihood statistics and data set from *Escherichia coli* we develop a robust method for neutralizing technical biases (e.g. base specific RNase preferences in ribosome-protected mRNA fragments (RPF) generation), which allows for correct estimation of translation times at single codon resolution. Furthermore, we validated the method with available datasets from *E. coli* treated with antibiotic to inhibit isoleucyl-tRNA synthetase, and two datasets from *Saccharomyces cerevisiae* treated with two RNases with distinct cleavage signatures. We demonstrate that our approach accounts for RNase cleavage preferences and provides bias-corrected translation times estimates. Our approach provides a solution to the long-standing problem of extracting reliable information about peptide elongation times from highly noisy and technically biased ribosome profiling spectra.

## INTRODUCTION

Ribosome profiling (or Ribo-Seq) couples cell-wide profiling of the positions of translating ribosomes on messenger (mRNA) at single codon resolution (1) with deep sequencing (2) and has provided new insights into regulation of protein synthesis across species (reviewed in (3–5)). The approach requires rapid arrest of mRNA translation followed by isolation of intact mRNA-ribosome complexes, nuclease digestion of unprotected mRNA and generation of a deep-sequencing library from the ribosome-protected mRNA fragments (RPFs) (2). Interpretation of the RPFs in terms of elongation times at single codon resolution requires (i) ribosomal arrest to be faster than the single peptide elongation steps, (ii) precise estimation of the distance of the ribosomal A site (that is the ribosomal site accepting aminoacyl-tRNA-elongation factor complex) from the 5′- or 3′-ends of RPFs, (iii) neutralization of sequence-dependent biases in the experimental protocol (i.e. nuclease cleavage, amplification in the library preparation) (3,6). Fulfillment of these criteria enables determining translation time for any particular codon in the transcriptome.

Codon resolution of the RPF spectra is generally higher in eukaryotes than in bacteria. In eukaryotes, RNase I is the nuclease of choice and it cleaves precisely at ribosome boundaries (7). RNase I is inhibited by the bacterial ribosome (8), thus micrococcal nuclease (MNase, S7 nuclease) is most widely applied in generating bacterial Ribo-Seq libraries. MNase, however, cleaves with base-dependent specificity, preferably before A and U (9). Systematic analysis reveals that the MNase generated RPFs have more variable lengths at their 5′- than at their 3′-ends (7,10). Consequently, using the more precise MNase cleavage at the 3′-end to infer the A-site codon position improves the resolution of bacterial ribosome profiling sets (6,7), yet the bias in RPF generation due to the nucleotide-dependent specificity of the MNase persists.

An additional source of bias in the Ribo-Seq libraries is the local RPF sequence composition including high propensity for secondary structure formation for some RNA fragments which can interfere with the reverse transcription priming and/or with the adaptor ligation (11,12). Attempts at considering the systematic biases across Ribo-Seq libraries (13) or using smoothing algorithms to reduce data variance in the presence of the inherent heterogeneous noise of the ribosome profiling data sets (14,15) significantly improve the ability to distinguish genuine ribosome pausing from technical artifacts introduced by the library construction. Yet, a simple and robust method for neutralizing technical biases and extracting factors that determine the large sequence context dependent variations in translation speed even at identical ribosomal A-site codons is missing.

In the present work, we develop a model that accounts for the local codon context-dependent variation of peptide elongation times and RPF generation/processing biases. In total, we use 915 context-defining parameters, which are estimated by fitting the model-predicted RPF spectra to the experimental, transcriptome-wide RPF spectra using non-linear regression with maximum likelihood (ML) statistics. We also consider ribosome profiling spectra at single nucleotide resolution with homogenous fragment size to identify and neutralize RPF generation/processing biases near the 5′- and 3′-fragment ends. Our results suggest that an inner local context of five codons, including those at the A, P and E sites, accounts for the ribosomal dwell time on each A-site codon of the transcriptome. This determination of the peptide elongation times provides a basis for a detailed understanding of the dynamics of protein synthesis in living cells.

## MATERIALS AND METHODS

### Ribo-Seq library generation

*Escherichia coli* B strain AS19 was grown in LB medium until the culture reached an OD_600_ of 0.5. Cells were harvested by flash freezing and libraries from biological replicates were prepared for ribosome profiling by direct ligation of the platform-specific sequences or adapters as described (16). Sequenced RPFs were quality trimmed using *fastx-toolkit* (0.0.13.2; quality threshold: 20), sequencing adapters were cut using *cutadapt* (1.8.3); minimal overlap: (1 nt) and uniquely mapped to the *E. coli* genome (strain MG1655, version U00096.3, NCBI) using Bowtie (1.2.2) with parameters -l 16 -n 1 -e 50 -m 1—strata—best y. The RPF counts for each ORF were normalized per total mapped reads per million (RPM) (17) and calibrated to the A site using the 3′-ends of the RPFs as described earlier (18). The data sets generated in this study are accessible under the accession number GSE145571. Furthermore, we analyzed in the same way the following data sets: GSM3358136 and GSM3358137 for Ribo-Seq libraries of *E. coli* MG1655 cultured in MOPS complete synthetic media containing all 20 amino acids with no treatment or treated for 10 min with 200 μM mupirocin, respectively, and collected by filtration (6), and GSM2186726 and GSM2186728 for *S. cerevisiae* libraries in which the RPFs were generated using MNase and RNase A, respectively (19).

### Modeling strategy for Ribo-Seq spectra

Each RPF is assigned to a codon position *j* of the open reading frame from gene *i*, ORF*_i_*. The detected number of RPFs, $c_{ij}^{\exp }$, often colloquially referred to as ‘RPF counts’, reflects the number of ribosomes with this particular codon in A site at the moment of flash-freezing of the cells as well as biases in the nuclease digestion of mRNA and in the further amplification/processing to DNA libraries (3,9,11,12,14,20). The expected value ${\lambda _{ij}}$ of the stochastic integer $c_{ij}^{\exp }$ at any A-site codon position (*i,j*) we write as:

(1) $$\begin{equation*}{\lambda _{ij}} = {c_{RPF}} \cdot {\nu _i} \cdot {\tau _{ij}} \cdot \gamma _{ij}^B.\end{equation*}$$

Here, ${c_{RPF}}$ is the same constant for all A-site positions (*i,j*), ${\nu _i}$ is the global frequency of translation initiation of an ORF of type *i* in the cell population and proportional to the ORF_*i*_ expression level, ${\tau _{ij}}$ is the expected peptide elongation cycle time, $\gamma _{ij}^B$ is a ‘bias’ factor that depends on the context of codon *j* in ORF*_i_* and reflects the extent of digestion/processing/ amplification biases in Ribo-Seq library preparation. We note that ${c_{RPF}}$ constant reflects the depth of Ribo-Seq library. Its numeric value depends on the number of translating ribosomes in the cell population used for library preparation and also on the efficiencies of ligation, RPF amplification and sequencing.

Each elongation time ${\tau _{ij}}$ in Equation (1) is the product of a time calibration factor ${\tau ^e}$ and a parameter $\gamma _{ij}^T$ that, like $\gamma _{ij}^B$, depends on the context of codon *j* but is proportional to the peptide elongation cycle time: ${\tau _{ij}} = {\tau ^e}\gamma _{ij}^T$. Accordingly, we re-write Equation (1) as:

(2) $$\begin{equation*}{\lambda _{ij}} = {c_{RPF}} \cdot {\nu _i} \cdot {\tau ^e} \cdot \gamma _{ij}^T \cdot \gamma _{ij}^B = {\varphi _i} \cdot {\gamma _{ij}}.\end{equation*}$$

Here, parameter ${\varphi _i} = {c_{RPF}}{\nu _i}{\tau _e}$ is proportional to global frequency of translation initiation ${\nu _i}$ of ORF*_i_* and ${\gamma _{ij}}$ is defined by ${\gamma _{ij}} = \gamma _{ij}^T \cdot \gamma _{ij}^B$. The expected value of RPF counts, ${\lambda _{ij}}$, contains two factors of great physiological relevance, namely the protein expression level ${\nu _i}$ from gene *i* and the expected peptide elongation cycle time ${\tau _{ij}} = {\tau ^e}\gamma _{ij}^T$ at A-site codon *j* in transcript *i*. The major methodological task is, therefore, to elicit reliable estimates of the expected values, ${\varphi _i}$, proportional to ${\nu _i}$, and ${\tau _{ij}}$ for all codons (*i*, *j*) from the experimental sets of the sampled $c_{ij}^{\exp }$ values and known growth rate, $\mu$, of the bacterial culture. When $c_{ij}^{\exp }$ is much larger than 1, it provides a reliable estimate of ${\lambda _{ij}}$ but for small $c_{ij}^{\exp }$ values its statistical nature must be accounted for by the probability $P(c_{ij}^{\exp })$ that the number of RPF counts from A-site codon (*i,j*) is $c_{ij}^{\exp }$. The RPF counts $c_{ij}^{\exp }$ are obtained from ligated RNA fragments with copy numbers amplified by PCR and greatly reduced in the sequencing procedure. The probability distributions for RNA fragments after ligation are of Poisson type (Supplementary Text), the distributions of DNA fragments after amplification are burst-like (21) and the distributions of sequenced DNA fragments ${P_{NA}}(c_{ij}^{\exp }|\lambda _{ij}^{},v = A \cdot q)$ are of Neyman type A. At small values $v = A \cdot q$, where *A* is the PCR copy number amplification factor and *q* is the fraction of the amplified library that has been finally sequenced, the Neyman type A distribution is close to Poisson but with variance equal to the expected value (${\lambda _{ij}}$) multiplied by a constant factor $1 + A \cdot q$ (Supplementary Text). Under our experimental conditions the $A \cdot q$ product is smaller than 1, and for the simplicity in what follows, we assume the copy number distribution for any (*i,j*) fragment to be of Poisson type:

(3) $$\begin{equation*}Po(c_{ij}^{\exp }|{\lambda _{ij}}) = \frac{{{{\left( {{\lambda _{ij}}} \right)}^{c_{ij}^{\exp }}}}}{{c_{ij}^{\exp }!}}{e^{ - {\lambda _{ij}}}}\end{equation*}$$

We ascribe a log-likelihood function *L* for the whole transcriptome based on all $Po(c_{ij}^{\exp }|{\lambda _{ij}})$ probabilities:

(4) $$\begin{equation*}L = {\log _e}\left( {\prod\limits_{i,j} {Po(c_{ij}^{\exp }|{\lambda _{ij}})} } \right).\end{equation*}$$

**Figure 1.**
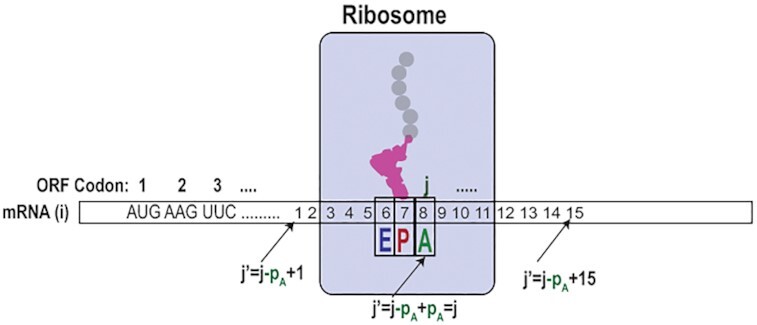
Local and global codon contexts for a ribosome translating an ORF of type *i*. Global A-site parameter *j* corresponds to the local A-site parameter *p = p_A_*= 8. Global parameter *j'* corresponds to the local parameter *p* through *j' = j-p_A_+p*, where *p* varies from 1 to *p_L_*= 15, so that P and E site correspond to *p*= 7 and *p* = 6, respectively.

In what follows we develop a model for the ${\lambda _{ij}}$ values in Equation 1 built on the hypothesis that each ${\gamma _{ij}}$ in Equation (2) is determined by a local context of the current A-site codon *j* in ORF*_i_* and that this context is composed of *p_L_*, codons with the A site at its near middle position (Figure 1). For clarity, below we make explicit three distinct description levels of parameters in our approach: directly experimental (e.g. $c_{ij}^{\exp }$), modelled (e.g. $c_{ij}^{\bmod }$) and expected (e.g. ${\lambda _{ij}}$) values of key parameters. For ease of identification, we also use the Latin letters for the first two categories and Greek letters for the third one (Table 1).

**Table 1.** Meaning of key parameters of the present work

Expected parameters	
${\lambda _{ij}} = {c_{RPF}} \cdot {\nu _i} \cdot \tau _{ij}^{} \cdot \gamma _{ij}^B = {\varphi _i}{\gamma _{ij}}$	Expected number of RPF counts from codons at position *j* in open reading frames from gene i (ORF*_i_*) in cell population
${c_{RPF}}$	Constant reflecting the depth of the Ribo-Seq library as determined by the number of translating ribosomes in the cell population, efficiencies of RPF generation, ligation, amplification and sequencing.
${\nu _i}$	Expected number of initiations on ORF*_i_*.
$\gamma _{ij}^B$	Technical factor, determined by context bias efficiencies of RPF generation, ligation and amplification for codon *j* in ORF*_i_*.
${\tau _{ij}} = \gamma _{ij}^T \cdot {\tau ^e}$	Expected elongation cycle time for codon *j* of ORF*_i_*.
${\tau ^e}$	Expected elongation cycle time average for the whole cell population.
$\gamma _{ij}^T$	Expected codon context dependent elongation cycle time for codon *j* of ORF*_i_* normalized to *τ*^e^.
${\varphi _i} = {c_{RPF}}{\nu _i}{\tau _e}$	Factor proportional to global frequency ${\nu _i}$ of translation initiation of ORF*_i_*.
$\gamma _{ij}^{} = \gamma _{ij}^T \cdot \gamma _{ij}^B$	Expected codon context dependent variation of number of RPFs normalized to factor ${\varphi _i}$ and partitioned into elongation cycle time and bias factors.
**Experimental parameters**	
$c_{ij}^{\exp }$	Measured number of RPF counts for A-site codon at position *j* in ORF*_i_*.
$C_i^{\exp } = \sum\nolimits_j {c_{ij}^{\exp }}$	Sum of RPF counts for the ‘inner’ region of ORF*_i_* containing $n_i^{}$ codons.
$d_i^{\exp } = C_i^{\exp }/n_i^{}$	Mean RPF density in the ‘inner’ ORF*_i_* region containing $n_i^{}$ codons.
$\Omega _{p,c}^{\exp } \equiv \sum\nolimits_i {} \sum\nolimits_j^{} {c_{ij}^{\exp }{\delta _{c,se{q_i}(j + p - {p_A})}}}$	Sum of $c_{ij}^{\exp }$ over all positions *j* in all ORFs for which there is a codon of type *‘c’* at position *‘j+p-p_A_’* .
$s_{ij}^{\exp } = {n_i} \cdot c_{ij}^{\exp }/C_i^{\exp } = c_{ij}^{\exp }/d_i^{\exp }$	RPF score function describing relative variation of $c_{ij}^{\exp }$ along ORF*_i_*.
**Modell parameters**	
$c_{ij}^{\bmod } = f_i^{\bmod } \cdot g_{ij}^{\bmod }$	Maximum likelihood (ML) estimate of λ_*ij*_.
$g_{ij}^{\bmod } = \prod\nolimits_{p = 1}^{{p_A}} {{z_{p,se{q_i}(j + p - {p_A})}}}$	ML estimate of ${\gamma _{ij}}$ by a number *p_L_* of ${z_{p,c}}$ factors, each with 64 codon identity determined values.
${z_{p,c}}$	Underlying parameters of our model, determined from the ML fit of all $c_{ij}^{\bmod }$ to all $c_{ij}^{\exp }$ values.
$f_i^{\bmod } = \frac{{C_i^{\exp }}}{{\sum\nolimits_j^{} {g_{ij}^{\bmod }} }}$	ML estimate of ${\varphi _i}$.
$C_i^{\bmod } = \sum\nolimits_j {c_{ij}^{\bmod }}$	ML estimate of the sum of model RPF counts for the ‘inner’ region of ORF_i_. From the expressions for $c_{ij}^{\bmod }$ and $f_i^{\bmod }$ it follows that $C_i^{\bmod } = C_i^{\exp }$.
$G_i^{\bmod } = \sum\nolimits_i^{} {g_{ij}^{\bmod }}$	ML estimate of$\sum\nolimits_i^{} {\gamma _{ij}^{}}$.
$s_{ij}^{\bmod } = {n_i} \cdot c_{ij}^{\bmod }/C_i^{\bmod } = {n_i} \cdot g_{ij}^{\bmod }/G_i^{\bmod }$	RPF score function describing relative variation of $c_{ij}^{\bmod }$ along ORF*_i_*.
$t_{ij}^{\bmod } = {t^e}g_{ij}^T$	Model estimate of the expected elongation cycle time ${\tau _{ij}}$ for codon *j* of ORF*_i_*.
${t^e}$	Model estimate of time factor ${\tau ^e}$; experimentally determined from the growth rate $\mu$ of cell population.
$g_{ij}^T = \prod\nolimits_{p = {p_1}}^{{p_2}} {{z_{p,se{q_i}(j + p - {p_A})}}}$	Model estimate of bias-free relative elongation cycle time for codon *j* of ORF*i;* determined by the product ${z_{p,c}}$ factor for inner position of local context of codon (*i,j*).
$G_i^T = \sum\nolimits_i^{} {g_{ij}^T}$	Model estimate of bias-free total time for ORF*_i_* translation normalized to *t^e^*.
$T_i^{\bmod } = \sum\nolimits_j^{} {t_{ij}^{\bmod }} = {t^e}G_i^T$	Model estimate of absolute total time for ORF*_i_* translation.
$s_{ij}^T = {n_i} \cdot g_{ij}^T/G_i^T$	Pausing score function describing relative variation of bias free translation time $t_{ij}^{\bmod }$ along ORF*_i_*.

### Ribo-seq spectral modeling at single codon resolution

To obtain estimates for all ${\lambda _{ij}}$ values (Eq. 1 or 2), we introduce model RPF counts, $c_{ij}^{\bmod }$ composed of a factor $f_i^{\bmod }$ for gene *i*, estimating ${\varphi _i}$ multiplied by a local context factor $g_{ij}^{\bmod }$, estimating ${\gamma _{ij}}$ in Equation (2):

(5) $$\begin{equation*}c_{ij}^{\bmod } = f_i^{\bmod } \cdot g_{ij}^{\bmod }.\end{equation*}$$

Each local codon context position (*p*) among the total number *p_L_* of context defining positions contributes with a factor ${z_{p,c}}$ to the value of $g_{ij}^{\bmod }$:

(6) $$\begin{equation*}g_{ij}^{\bmod } = \prod\limits_{p = 1}^{{p_L}} {} {z_{p,se{q_i}(j + p - {p_A})}}.\end{equation*}$$

Each factor ${z_{p,c}}$ is determined by the identity (*c*) of each one of the 64 possible codons at each position *p* (Figure 1). Index $se{q_i}(j + p - {p_A})$ identifies the codon at local position *p*, corresponding to global codon position *j +* *p −p*_A_ in ORF*_i_* sequence (Figure 1). We fit the model RPF counts, $c_{ij}^{\bmod }$, to the experimental RPF counts, $c_{ij}^{\exp }$, in the inner ORFs regions, by adjusting the ${p_L} \times 61$ context factors ${z_{p,c}}$ to maximize a Poisson-based likelihood function (Equation 4). If not stated otherwise, we use *p_L_* = 15, so that a total of 915 (15 × 61 = 915) factors ${z_{p,c}}$ estimate all $g_{ij}^{\bmod }$-values in all potential contexts, where 61 is the number of sense codons. The *E. coli* transcriptome may contain up to 1.8 × 10^6^ distinct contexts (about 6000 ORFs and 300 codons per ORF), and the ultimate number of contexts for which $g_{ij}^{\bmod }$ could be predicted by the model is 61^15^$ \approx$ 10^27^. In the next section, we describe how model parameters are derived from experimental data by maximizing a transcriptome-wide log likelihood function.

### Ribo-seq spectral modeling with maximum likelihood (ML) estimation of local codon context parameters

To extract model parameters $f_i^{\bmod }$ (Equation 5) and ${z_{p,c}}$ (Equation 6) from Ribo-Seq datasets, we assume that each $c_{ij}^{\exp }$ value is sampled from a Poisson distribution with expected value ${\lambda _{ij}}$ (Equation 3), the latter estimated by the model parameter $c_{ij}^{\bmod }$ (Equations 5 and 6). The log-likelihood function *L* for the RPF spectrum takes the simple form (see also Equation 4):

(7) $$\begin{eqnarray*}L &=& \ln \prod\limits_{i,j} {Po(c_{ij}^{\exp }|c_{ij}^{\bmod })} \nonumber\\ &=& \sum\nolimits_i {\sum\nolimits_{j = {p_A}}^{j = {l_i} - {p_L} + {p_A}} {( - c_{ij}^{\bmod } + c_{ij}^{\exp }\ln c_{ij}^{\bmod } - \ln (c_{ij}^{\exp }!))} } .\nonumber\\ \end{eqnarray*}$$

Here, the *j*-summations for each ORF_*i*_ are confined to an internal ORF region starting at codon *p_A_* and ending at codon ${l_i} - {p_L} + {p_A}$ with a total number of internal codons, ${n_i} = {l_i} - {p_L}$, where ${l_i}$ is the total number of ORF_*i*_ codons. In what follows we use the short hand notation$\sum\nolimits_j { = \sum\nolimits_{j = {p_A}}^{j = {l_i} - {p_L} + {p_A}} {} }$ for the j-summations in Equation 7. The maximal value of *L* (Equation 7) is obtained by setting its partial derivatives with respect to all $f_i^{\bmod }$ and ${z_{p,c}}$ parameters equal to zero, which leads to the following equation system for determination of all ${z_{p,c}}$ parameters (see Supplementary Text):

(8) $$\begin{equation*}\Omega _{p,c}^{\exp } = \sum\nolimits_i {f_i^{\bmod }} \sum\nolimits_j {g_{ij}^{\bmod }{\delta _{c,se{q_i}(p + j - {p_A})}}} ,\end{equation*}$$

where

(9) $$\begin{equation*}f_i^{\bmod } = \frac{{C_i^{\exp }}}{{\sum\nolimits_j {g_{ij}^{\bmod }} }}.\end{equation*}$$

Here, ${\delta _{c,s}}$ is the ‘Kronecker delta function’ equal to 1 and 0, when $c = s$ and $c \ne s$, respectively; $C_i^{\exp } = \sum\nolimits_j^{} {c_{ij}^{\exp }}$ (Table 1) and $\Omega _{p,c}^{\exp }$ is a function that depends on codon type ‘*c*’ at local position ‘*p*’ (Figure 1). $\Omega _{p,c}^{\exp }$ is calculated from experimental RPF counts $c_{ij}^{\exp }$ (and sequence data) as:

(10) $$\begin{equation*}\Omega _{p,c}^{\exp } \equiv \sum\nolimits_i {} \sum\nolimits_j^{} {c_{ij}^{\exp }{\delta _{c,se{q_i}(j + p - {p_A})}}} .\end{equation*}$$

We note that in the special case *p = p*_A_ (Figure 1), $\Omega _{{p_A},c}^{\exp }$ is the total number of RPFs in a dataset generated by ribosomes with A-site codon of type ‘*c’*. More generally, $\Omega _{p,c}^{\exp }$ is the total number of RPFs for which there is a codon ‘*c’* at a distance $p - {p_A}$ from the A site.

With the help of Equation 6 that relates $g_{ij}^{\bmod }$ with ${z_{p,c}}$, Equations (8) and (9) are solved using a Levenberg-Marquardt type algorithm (21,22) to obtain the table of ${z_{p,c}}$ factors (see Supplementary Text). Using the obtained ${z_{p,c}}$ factors we compute local context parameters $g_{ij}^{\bmod }$ (Equation 6), and then model RPF counts $c_{ij}^{\bmod }$ from Equations (5) and (9) as:

(11) $$\begin{equation*}c_{ij}^{\bmod } = C_i^{\exp }\frac{{g_{ij}^{\bmod }}}{{\sum\nolimits_k {g_{ik}^{\bmod }} }} .\end{equation*}$$

Instead of comparing experimental ($c_{ij}^{\exp }$) and modelled ($c_{ij}^{\bmod }$) RPF spectra of the same transcript, it is more convenient to compare experimental ($s_{ij}^{\exp }$) and modeled ($s_{ij}^{\bmod }$) RPF scores defined here as:

(12) $$\begin{equation*}s_{ij}^{\exp } = {n_i}\frac{{c_{ij}^{\exp }}}{{\sum\nolimits_k {c_{ik}^{\exp }} }} \end{equation*}$$

and

(13) $$\begin{equation*}s_{ij}^{\bmod } = {n_i}\frac{{c_{ij}^{\bmod }}}{{\sum\nolimits_k^{} {c_{ik}^{\bmod }} }} = {n_i}\frac{{g_{ij}^{\bmod }}}{{\sum\nolimits_k {g_{ik}^{\bmod }} }},\end{equation*}$$

where *n_i_* is the total number of internal codons in ORF*_i_*.

The average RPF density of a gene:

(14) $$\begin{equation*}d_i^{\exp } = \frac{1}{{{n_i}}}\sum\nolimits_k^{} {c_{ik}^{\exp }} \end{equation*}$$

is often used as a statistical reliability measure of its RPF coverage profile.

The lower the $d_i^{\exp }$- value, the less informative the profile. For example, when $d_i^{\exp } < 0.5$ RPFs per codon, more than a half of the $c_{ij}^{\exp }$ values in the gene profile are zeroes and, hence, contain little information about codon translation times. We note that similar to *j*-summations above, the *k*-summations in Eqs. 11–14 are from *k* = *p_A_* to $k = {l_i} - {p_L} + {p_A}$ (Figure 1). We also note that experimental RPF scores $s_{ij}^{\exp }$ are sometimes referred to as ‘normalized footprint counts’ (23) or ‘relative enrichment values’ (24) and describe how much RPF counts for codon *j* deviate from a per-codon average value $d_i^{\exp }$ in the inner region of a gene.

The ${z_{p,c}}$ factors can always be scaled so that for each position $p = 1,....{p_L}$ of the local context we have (Supplementary Text):

(15) $$\begin{equation*}{\bar{z}_p} = \sum\nolimits_c^{} {{w_{p,c}}{z_{p,c}}} = 1,\end{equation*}$$

where the ${w_{p,c}}$ weighting factors are calculated as:

(16) $$\begin{equation*}w_{p,c}^{} = \frac{{\sum\nolimits_i {f_i^{\bmod }{n_{i,p,c}}} }}{{\sum\nolimits_i {f_i^{\bmod }{n_i}} }},\end{equation*}$$

and ${n_{i,p,c}}$ is :

(17) $$\begin{equation*}{n_{i,p,c}} = \sum\nolimits_j^{} {{\delta _{c,seq(p + j - {p_A})}}} .\end{equation*}$$

Since $f_i^{\bmod }$ estimates ${\nu _i}$, a parameter proportional to the expression level of gene *i* (Equation 1) and ${n_{i,p,c}}$ (Equation 17) varies little with position *p* (see Supplementary Text), each product $f_i^{\bmod }{n_{i,p,c}}$ in Equation 16 is proportional to the frequency with which the ribosome encounters a codon of a type *c* in the inner region of ORF*_i_*. Hence, each ${w_{p,c}}$ confers a statistical weight proportional to the frequency with which the ribosome encounters a codon of type *c* in the transcriptome (Supplementary Text).

We also introduce the ‘sensitivity parameter’ ${S_p}$ as a measure of the sensitivity of ${z_{p,c}}$ to the codon identity *c* at local context position *p*. It is defined as the standard deviation, ${\sigma _p}$, from the mean ${\bar{z}_p} = 1$ (Equation 15) for row *p* of the table of ${z_{p,c}}$ factors:

(18) $$\begin{equation*}{S_p} \equiv \sigma _p^{} = \sqrt {\sum\nolimits_c^{} {} {w_{p,c}}{{({z_{p,c}} - {{\bar{z}}_p})}^2}} ,\end{equation*}$$

where the weights ${w_{p,c}}$ are defined in Equation (16).

### Ribo-seq spectral modeling at single nucleotide resolution

In order to estimate the fragment processing bias ($\gamma _{ij}^B$; Equation 1), we extend our modeling resolution from codon to nucleotide level. For this, we use the number of RPFs, $c_{ij}^{\exp ,FL}$, of single length FL with ribosomal A site located at nucleotide *j* in ORF_*i*_ and estimate its expected value, $\lambda _{ij}^{FL}$ (compare with Equations 1 and 5) as:

(19) $$\begin{equation*}c_{ij}^{\bmod ,FL} = f_i^{\bmod ,FL} \cdot g_{ij}^{\bmod ,FL},\end{equation*}$$

where each parameter $g_{ij}^{\bmod ,FL}$ is modeled as product of local context z-factors (compare with Equation 6):

(20) $$\begin{equation*}g_{ij}^{\bmod ,FL} = \prod\limits_{p = 1}^{{p_{NL}}} {} z_{p,se{q_i}(j + p - {p_{NA}})}^{FL}.\end{equation*}$$

Here, index *j* in Equations (19) and (20) refers to nucleotide *j* of ORF*_i_*, and $se{q_i}(j)$ specifies nucleotide base *b* (U, C, A or G) at transcriptome position (*i,j*); $z_{p.b}^{FL}$ factors form a p_N_*_L_*x4 table; the local nucleotide position *p* is counted from *p= 1*, via the first nucleotide at A-site position *p = p_NA_* to the third base of the last codon of the local context sequence of length *p_NL_* (Supplementary Figure S1).

Parameters $z_{p.b}^{FL}$ are ML estimated by non-linear model fitting to experimental data assuming Poisson distributed RPF counts $c_{i,j}^{\exp ,FL}$. The data treatment is formally equivalent to that leading up to Equations. 8 and 9 with parameters $f_{ij}^{\bmod }$ and $\Omega _{p,c}^{\exp }$ replaced by$f_{ij}^{\bmod ,FL}$ and $\Omega _{p,b}^{\exp ,FL}$, respectively. Thus:

(21) $$\begin{equation*}\Omega _{p,b}^{\exp ,FL} = \sum\nolimits_i {} \frac{{C_i^{\exp }}}{{\sum\nolimits_j {} g_{ij}^{\bmod ,FL}}}\sum\nolimits_j {} g_{ij}^{\bmod ,FL}{\delta _{b,se{q_i}(p + j - {p_{NA}})}},\nonumber\\ \end{equation*}$$

where ${\delta _{b,s}}$ is the Kronecker delta function and $\Omega _{p,b}^{\exp ,FL}$ is obtained from experimental data $c_{i,j}^{\exp ,FL}$ through (compare with Equation 10) :

(22) $$\begin{equation*}\Omega _{p,b}^{\exp ,FL} = \sum\nolimits_i {{{\sum\nolimits_j^{} {c_{i,j}^{\exp ,FL}{\delta } _{b,se{q_i}(j + p - {p_{NA}})}}}}} .\end{equation*}$$

Assuming *x* to be the distance from the first A-site nucleotide to the 3′-end of the RPF in nucleotides, it follows that $\Omega _{{p_{NA}} + x - FL,b}^{FL}$ and $\Omega _{{p_{NA}} + x,b}^{FL}$ are the numbers of RPFs of length *FL* with nucleotide ‘*b*’ at 5′- and 3′-end, respectively. By applying the same ML procedure as in the codon-resolution case, we solve Equation 21 to estimate the $z_{p.b}^{FL}$ and $f_i^{\bmod ,FL}$ factors for computing all $g_{ij}^{\bmod ,FL}$ and $c_{ij}^{\bmod ,FL}$ parameters. Using formulae analogous to those in Eqs 12 and 13, one can compute the model nucleotide RPF scores $s_{ij}^{\exp ,FL}$ to compare them with the experimental scores $s_{ij}^{\bmod ,FL}$ for RPF profiles generated from RPFs with a length of FL nucleotides.

### Construction of unbiased Ribo-Seq spectra for estimation of relative peptide elongation times

To separate the effects of bias and peptide elongation time variations on the RPF counts, we partition the context dependent factors $g_{ij}^{\bmod }$ in Equation 5 into two parts:

(23) $$\begin{equation*}g_{ij}^{\bmod } = g_{ij}^Bg_{ij}^T,\end{equation*}$$

where (compare with Equation 6):

(24) $$\begin{equation*}g_{ij}^B = \prod\limits_{p = 1}^{{p_1} - 1} {} z_{p,se{q_i}(p + j - {p_A})}^{} \cdot \prod\limits_{p = {p_2} + 1}^{{p_L}} {} z_{p,se{q_i}(p + j - {p_A})}^{},\end{equation*}$$

and:

(25) $$\begin{equation*}g_{ij}^T = \prod\limits_{p = {p_1}}^{{p_2}} {{z_{p,se{q_i}(p + j - {p_A})}}} .\end{equation*}$$

As shown in Results, the outer context dependent factors $g_{ij}^B$, determined by $z_{p,c}^{}$ factors for outer local context positions *p* from 1 to *p_1_*–1 and from *p_2_*+1 to *p_L_*, mainly account for the nuclease digestions/processing biases (*B*). The inner context dependent factors $g_{ij}^T$, determined by $z_{p,c}^{}$ factors for inner positions *p* (from *p_1_* to *p_2_*), mainly reflect the variation of the peptide elongation time, hence superscript (*T*) in $g_{ij}^T$. We model the bias-free RPF spectrum as:

(26) $$\begin{equation*}c_{ij}^T = f_i^{\bmod }g_{ij}^T.\end{equation*}$$

We also introduce model pausing scores $s_{ij}^T$ to quantify the *relative* peptide elongation time as the ribosome moves along an ORF*_i_* (compare with Equations 12 and 13):

(27) $$\begin{equation*}s_{ij}^T = {n_i}\frac{{g_{ij}^T}}{{\sum\nolimits_k^{} {g_{ik}^T} }}.\end{equation*}$$

From the 15 $z_{p,c}^{}$ factors used to model the experimental $c_{ij}^{\exp }$ values in the dataset we normally use five inner $z_{p,c}^{}$ factors to obtain bias-corrected model RPF counts $c_{ij}^T$ (Eqs 25, 26). This approach is distinct from using a $\tilde{z}_{p,c}^{}$-parameter function $\tilde{g}_{ij}^{\bmod }$, defined only by the inner codons of the local context in the *p*-interval from *p_1_* to *p_2_*:

(28) $$\begin{equation*}\tilde{g}_{ij}^{\bmod } = \prod\limits_{p = {p_1}}^{{p_2}} {{{\tilde{z}}_{p,se{q_i}(p + j - {p_A})}}} .\end{equation*}$$

When the ML method is used to estimate the *inner*$\tilde{z}_{p,c}^{}$ parameters in Equation 28 that best account for the *whole* RPF spectrum, strong technical biases inherent to $c_{ij}^{\exp }$ spectra distort the $\tilde{z}_{p,c}^{}$ factors. This makes the model RPF scores $\tilde{s}_{ij}^{\bmod }$, defined as:

(29) $$\begin{equation*}\tilde{s}_{ij}^{\bmod } = {n_i}\frac{{\tilde{g}_{ij}^{\bmod }}}{{\sum\nolimits_k {\tilde{g}_{ik}^{\bmod }} }},\end{equation*}$$

distinct from and inferior to the more accurate elongation time estimating pause scores $s_{ij}^T$ (Equation 27).

### Absolute peptide elongation cycle times from exponential growth rate

The expected time, ${\tau _{ij}}$, to translate a codon at position *j* of gene *i* in the cell (Equation 1) is estimated by the model time, $t_{ij}^{\bmod }$, defined by the product of the local context factor $g_{ij}^T$ (Equation 25) and a time factor ${t^e}$, estimating ${\tau ^e}$ in Equation 1:

(30) $$\begin{equation*}t_{ij}^{\bmod } = {t^e}g_{ij}^T.\end{equation*}$$

It follows that the total expected time *T_i_* to translate ORF*_i_* (Table 1) is estimated by:

(31) $$\begin{equation*}T_i^{\bmod } = \sum\nolimits_j^{} {t_{ij}^{\bmod }} = {t^e}G_i^T,\end{equation*}$$

where

(32) $$\begin{equation*}G_i^T = \sum\nolimits_j^{} {g_{ij}^T} .\end{equation*}$$

We note that the $G_i^T$ value estimates relative translation time of protein *i* (Table 1). Let *P_i_* be the number of proteins of a type *i* in an exponentially growing cell population at a given time. The rate of copy number increase for proteins of a type ‘*i*’ is:

(33) $$\begin{equation*}\frac{d}{{dt}}{P_i} = \gamma P_R^{}\frac{{{u_i}}}{{T_i^{\bmod }}} .\end{equation*}$$

Here, ${P_R}$ is the current number ribosomes in the population, $\gamma$ the fraction of ribosomes in elongation phase, estimated as 0.8 by Dennis and Bremer (25) and ${u_i}$ is the fraction of elongating ribosomes devoted to synthesis of protein *i*. Fraction *u_i_* is proportional to the sum, $C_i^T$, of bias-corrected RPF counts $c_{ij}^T$ for ORF*_i_*. Taking Equations 26 and 32 into account one gets for $C_i^T$:

(34) $$\begin{equation*}C_i^T = f_i^{\bmod }G_i^T,\end{equation*}$$

so that ${u_i} = C_i^T/C_{tot}^T$, where $C_{tot}^T = \sum\nolimits_i {C_i^T}$. Using this and Equations 31 and 34, one can re-write Equation 33 as:

(35) $$\begin{equation*}\frac{d}{{dt}}{P_i} = \frac{{\gamma {P_R}}}{{C_{tot}^T}}\frac{{C_i^T}}{{T_i^{\bmod }}} = \frac{{\gamma {P_R}}}{{C_{tot}^T{t_e}}}f_i^{\bmod } .\end{equation*}$$

Introducing ${P_{tot}} = \sum\nolimits_{}^{} {{P_i}}$, the sum total of current protein copies, the exponential growth rate, $\mu$ can be defined as the increase in total protein copy number per time unit ($d{P_{tot}}/dt$) normalized to ${P_{tot}}$:

(36) $$\begin{equation*}\mu = \frac{1}{{{P_{tot}}}}\frac{{d{P_{tot}}}}{{dt}} .\end{equation*}$$

We note that for exponential growth the above definition of growth rate (Equation 36) is equivalent to its standard definition (26) as the rate of relative increase in total protein mass (see Supplementary Text). Taking Equations (34) and (35) into account, Equation (36) for the growth rate becomes:

(37) $$\begin{equation*}\mu = \frac{\gamma }{{{t_e}}}\frac{{{P_R}}}{{{P_{tot}}}}\frac{1}{{C_{tot}^T}}\sum\nolimits_i {f_i^{\bmod }} = \frac{\gamma }{{{t_e}}}\frac{{f_R^{\bmod }}}{{\sum\nolimits_i {G_i^T} f_i^{\bmod }}} .\end{equation*}$$

Here, we used that during exponential growth and when the rate of protein degradation is negligible compared to growth rate, the protein copy numbers ${P_i}$ are proportional to our estimates, $f_i^{\bmod }$, of the frequencies, ${\nu _i}$ (Equation 1) of protein *i* translation initiation, so that the relation ${P_R}/{P_{tot}} = f_R^{\bmod }/\sum\nolimits_i {f_i^{\bmod }}$ is valid (see Supplementary Text). From Equation (37) one obtains:

(38) $$\begin{equation*}{t^e} = \frac{\gamma }{\mu }\frac{1}{{\sum\nolimits_i^{} {G_i^T(f_i^{\bmod }/f_R^{\bmod })} }},\end{equation*}$$

so that the model time, $t_{ij}^{\bmod }$ is

(39) $$\begin{equation*}t_{ij}^{\bmod } = \frac{\gamma }{\mu }\frac{{g_{ij}^T}}{{\sum\nolimits_k^{} {G_k^T(f_k^{\bmod }/f_R^{\bmod })} }} .\end{equation*}$$

Note that all parameters in Equations 38 and 39 except $\mu$ and $\gamma$ can be obtained from the Ribo-seq experiments themselves. The time factor ${t^e}$ in Equation 38 can be interpreted as an average per codon elongation time for a particular growth condition of the cell population, conditional on our special scaling of ${z_{p,c}}$ parameters (Equation 15) which forces $g_{ij}^T$ in Equation (30) to oscillate around 1. Importantly, despite that both ${t^e}$ and $g_{ij}^T$ depend on ${z_{p,c}}$ scaling, their product, the model time $t_{ij}^{\bmod }$, is scaling insensitive and estimates the absolute time $\tau _{ij}^{}$ of codon (*i,j*) translation (see Equation 39).

### Self-consistency of the RPF spectrum modeling

By self-consistent modeling we mean that a parameter estimation procedure applied to a dataset simulated using parameters extracted from the original data, will produce exactly the same parameter values as determined directly from the original data. It can be proven that our procedure of extracting the underlying parameters ${z_{p,c}}$ is indeed self-consistent (see also Supplementary Text). To illustrate this, we first use our ML approach to estimate an original ${z_{p,c}}$ parameter table from experimental RPF data, then use Equations (6) and (11) to simulate an RPF dataset and, finally, retrieve a new ${z_{p,c}}$ parameter table from the simulated RPF dataset. We find that the original and retrieved ${z_{p,c}}$ parameter tables are virtually identical as illustrated in Supplementary Figure S2A for A-, P- and E-site positions of ${z_{p,c}}$ parameter tables. In contrast, other methods like RUST (14) are not self-consistent in this sense. Computing the RUST ratio metafile table to simulate RPF data and then applying RUST again to retrieve the RUST ratio metafile one finds that the original and retrieved metafile tables differ significantly as illustrated for A-, P- and E-site metafile positions in Supplementary Figure S2B.

## RESULTS

### Modeling of Ribo-Seq spectra

**Figure 2.**
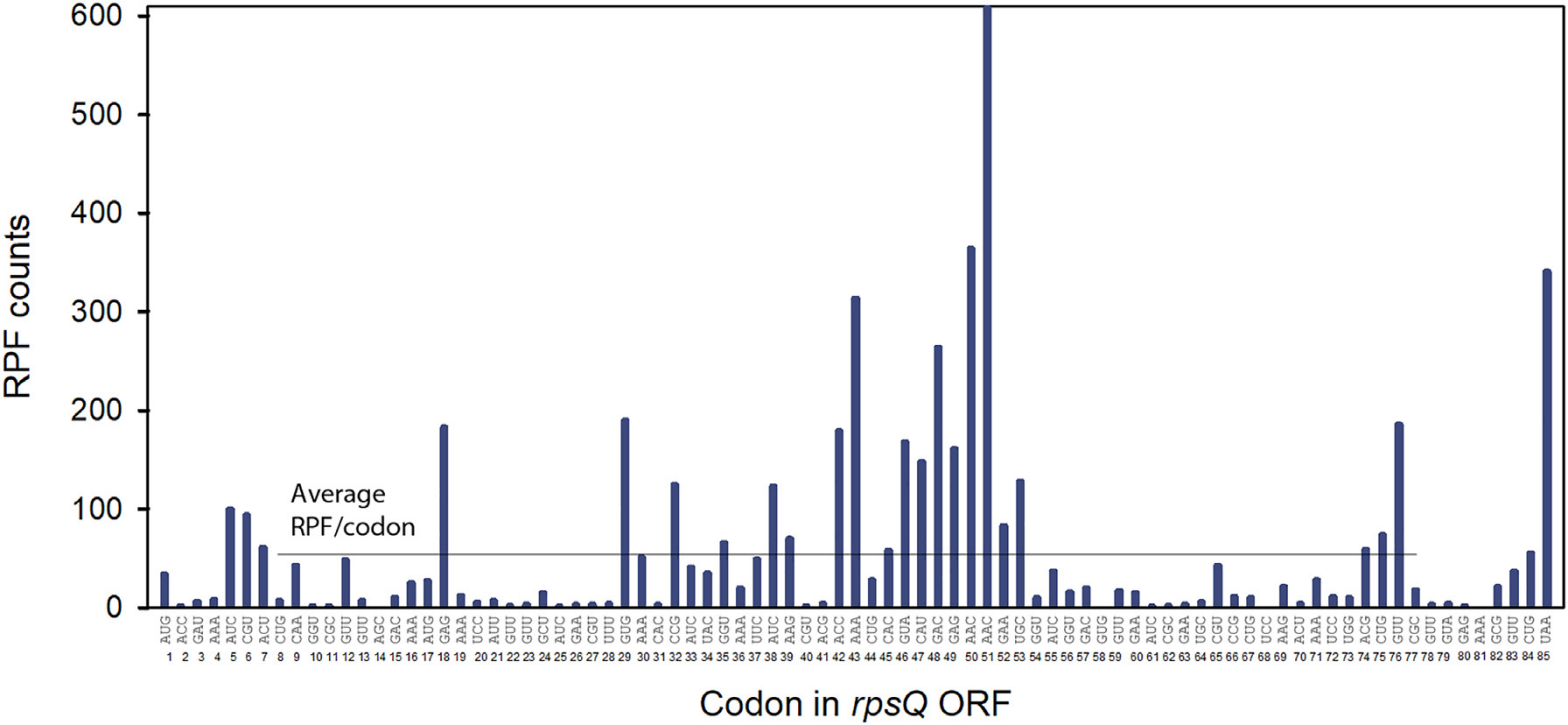
Ribosome profiling spectrum for gene *rpsQ*. RPF counts ($c_{ij}^{\exp }$-values) are plotted versus codon position *j* of the *rpsQ* transcript encoding ribosomal protein S17. The horizontal line represents the average number of RPFs per codon ($d_i^{\exp } = 61.5$) for the inner transcript region from ${j_S} = 8$ to ${j_E} = 77$ (see Equation 14 for formal $d_i^{\exp }$ definition).

There is a clear connection between the expected number, ${\lambda _{ij}}$, of experimentally detected ribosomes with a particular codon *j* of ORF*_i_* in the A site, and the expected codon translation time ${\tau _{ij}}$ (Equation 1). This connection allows one to use ribosome profiling for transcriptome-wide kinetic analysis of mRNA translation, but attainment of reliable kinetics data from ribosome profiling has remained elusive. The codon coverage within ORFs in the ribosome profiling spectra is highly variable (Figure 2). This is not only due to the codon context dependent variation of the codon translation time but also to context-dependent bias in the efficiency of nuclease dependent RPF generation and subsequent DNA library preparation steps including reverse transcription, adaptor ligation and PCR (9,11,12,14,20,24,26,27). Here, we consider three major causes of codon-to-codon variation of the experimental (‘exp’) RPF counts $c_{ij}^{\exp }$ at each transcriptome position (*i, j*) summarized in Equation 1. These include: (i) codon context-dependent variation in the peptide elongation time, ${\tau _{ij}}$, (ii) bias, $\gamma _{ij}^B$, of RPF generation and processing, and (iii) stochastic fluctuations in the experimental $c_{ij}^{\exp }$ values. As seen in Equation (2), each ${\tau _{ij}}$ value is the product of a time factor ${\tau ^e}$ reflecting average codon translation time under a particular growth condition and a unit-less parameter $\gamma _{ij}^T$ that depends on the context of codon *j*, so that ${\tau _{ij}} = {\tau ^e}\gamma _{ij}^T$. Local context dependent variation of $\gamma _{ij}^T$ that causes the variations in ${\tau _{ij}}$ can be traced to identities of A-, P- and E-site tRNAs, interactions between mRNA codons and the ribosome and/or interactions of the nascent peptide chain with the ribosomal exit tunnel in an amino acid-sequence dependent manner (28–30). The variations of bias factor $\gamma _{ij}^B$ are also due to local context dependence of the nuclease digestion and/or amplification/processing steps in RPF library preparation. From these, it follows that the variation of the product ${\gamma _{ij}} = \gamma _{ij}^T \cdot \gamma _{ij}^B$ that reports on variations of expected counts, ${\lambda _{ij}}$ (Equation 2), is defined by local sequence context of the current A-site codon *j* in ORF*_i_* (Figure 1).

**Figure 3.**
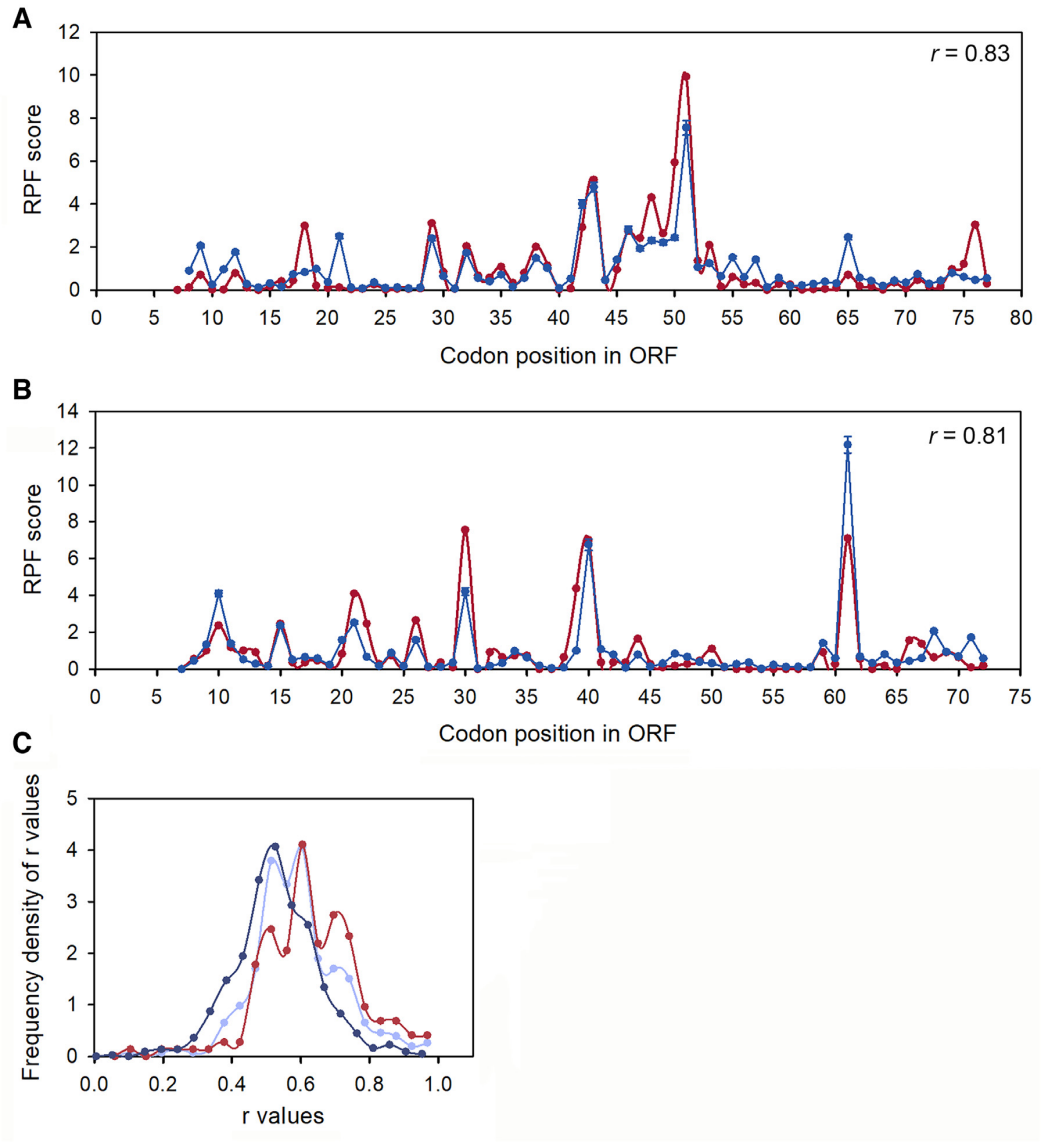
Comparisons of experimental ($s_{ij}^{\exp }$; red; Equation 12) and model ($s_{ij}^{\bmod }$; blue; Equation 13) RPF score spectra at codon resolution for *rpsQ* (**A**) and *atpE* transcript (**B**). *r*, Pearson correlation, *r* = 0.83 for *rpsQ* and *r* = 0.81 for *atpE*. (**C**) Frequency density of Pearson correlation coefficient, *r*, between $s_{ij}^{\exp }$ and $s_{ij}^{\bmod }$ for sets of 161 (red, $d_i^{\exp } >5$), 337 (light blue, $d_i^{\exp } >1.5$) and 945 (dark blue, $d_i^{\exp } >0.3$) transcripts. Note that the transcripts were first ranked by their *d^exp^*-values (Figure 2) and then top-ranked 161, 337 and 945 transcripts were considered.

We estimated each ${\gamma _{ij}}$ value by a model (‘mod’) $g_{ij}^{\bmod }$ parameter, which is the product of 15 ${z_{p,c}}$ factors (Equation 6). Each *z*_*p,c*_ value is determined by the type of codon (*c*) at local sequence context position (*p*) (Figure 1). These ${z_{p,c}}$ values were estimated by fitting our model (Equations 5 and 6) to the experimental $c_{ij}^{\exp }$ values of the whole transcriptome. To illustrate the goodness of the fit, we compare experimental (Equation 12) and model (Equation 13) RPF scores for single genes with high RPF density. The model-predicted, $s_{ij}^{\bmod }$, and experimental, $s_{ij}^{\exp }$, RPF score spectra show relative codon-to-codon variation of modeled and experimental RPF counts. They can be remarkably similar at the single gene level (Figure 3A, B) with Pearson correlation coefficients, *r*, in the 0.7–0.8 range, suggesting that the local mRNA sequence context accounts for the major part of the variability of experimental $c_{ij}^{\exp }$ values. Figure 3C shows that high *r*-values are frequent for genes with high experimental RPF density. The *r* -values decrease as an increasing number of genes with medium and low RPF density are included in the comparison – an effect due to the high statistical uncertainty of RPF profiles for genes with low experimental RPF density. In comparison with the RUST method (14), our method achieves, on average, significantly higher Pearson correlations between experimental and model RPF spectra (Supplementary Figure S3).

### Ribosomal profiling spectra are ultra-sensitive to codon identity near ribosome edges

**Figure 4.**
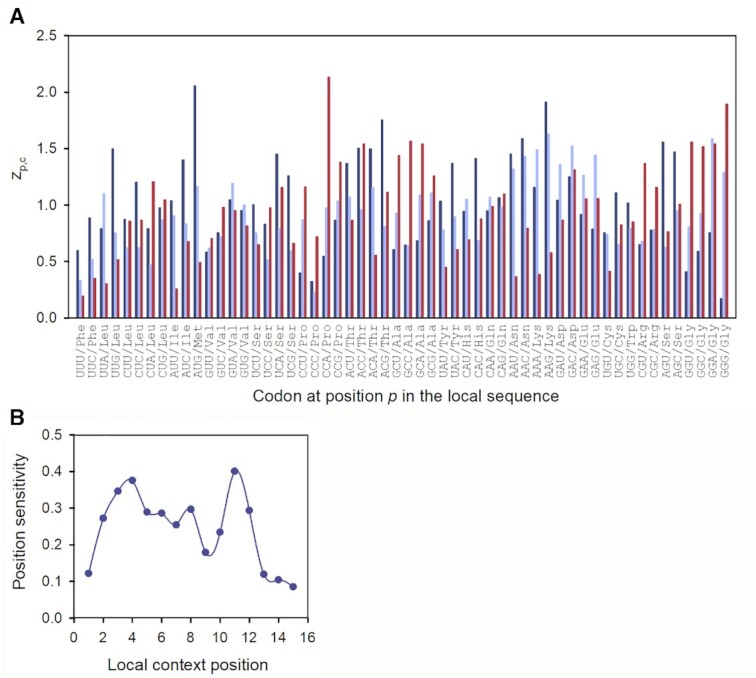
Sensitivity of ${z_{p,c}}$ factor with codon identity at different local context positions *p*. (**A**) Variation of ${z_{p,c}}$ values with codon identity *c* for positions *p* = 4 (dark blue, lagging ribosome edge), *p* = 8 (light blue, A site) and *p* = 11 (red, leading ribosome edge). Codons are ordered as in the genetic code table. (**B**) Position sensitivity *S_p_* (Equation 18) versus local context position *p* (see Figure 1 for position numbering).

Strikingly, variation of the ${z_{p,c}}$ factors with codon identity *c* is much larger for local codon positions (*p*) near the lagging (*p* = 4) and leading (*p* = 11) ribosome edges than in A site (*p* = 8) (Figure 1). Indeed, the ${z_{8,c}}$ value varies from 0.4 for the UUU (Phe) codon to 1.6 for the AAG (Lys) codon, while ${z_{4,c}}$ and ${z_{11,c}}$ values span significantly larger ranges from 0.2 for the GGG (Gly) to 2.1 for the AUG (Met) codon for ${z_{4,c}}$ and from 0.2 for the UUU (Phe) to 2.2 for the CCA (Pro) codon for ${z_{11,c}}$, respectively (Figure 4A). We have quantified the sensitivity of ${z_{p,c}}$ to codon identity *c* at position *p* as a weighted standard deviation, *S_p_*, from the mean along the *p*-row of the ${z_{p,c}}$-factor table (Equation 18). A plot of *S_p_* versus *p* confirms much higher sensitivity to codon identity at local codon positions close to ribosome edges (*p* = 4 and *p* = 11) than at ribosomal A, P or E site (*p* = 8, 7 or 6, respectively) (Figure 4B).

### Nuclease induced bias in Ribo-Seq spectra from *E. coli*

To dissect the origins of enhanced codon sensitivity of ${z_{p,c}}$ factors at positions near ribosome edges (Figure 4), we analyzed Ribo-Seq spectra also at single nucleotide resolution. Bacterial Ribo-Seq libraries are commonly constructed by first mapping the 3′-ends of RPFs to genomic nucleotide sequences (6,7). RPF coverage profiles at single nucleotide resolution are then obtained by counting the number $\tilde{c}_{ij}^{\exp }$ of RPFs assigned to nucleotide *j* of gene *i*. The $\tilde{c}_{ij}^{\exp }$-values are subsequently converted to standard experimental RPF profiles at single nucleotide resolution $c_{i,j}^{\exp }$ by the re-assignment rule $c_{i,j}^{\exp } = \tilde{c}_{i,j + x}^{\exp }$. The premise for this procedure is that the nucleotide distance (*x*) from the 3′-end to the first A-site nucleotide of an RPF is constant (6). Fragment length(FL)-specific profiles, $c_{i,j}^{\exp ,FL}$, are generated from RPFs of the same length, FL, so that the standard $c_{i,j}^{\exp }$ profiles can also be obtained by summation of $c_{i,j}^{\exp ,FL}$ over all FLs. In bacteria, both FL-summed and single FL-specific RPF coverage profiles lack the well-defined three-nucleotide periodicity that is observed in yeast or mammalian cells (6,7). We suggest that this periodicity loss is caused by ‘anomalous’ MNase cleavage at one or two nucleotides downstream of the ordinary cleavage site at the leading (3′) edge of the ribosome. Consequently, the RPF profiles appear as if the translating ribosome moves one nucleotide at a time. In both, single-codon resolution (Equation 1) and single-nucleotide resolution cases, there are expected numbers of RPFs, $\lambda _{ij}^{FL}$, generated from ribosomes with their A site at nucleotide number *j* of ORF*_i_*. The local 15-codon context (*p_L_* = 15) with the A-site codon at position *p_A_* = 8 (Figure 1) here corresponds to a local 45-nucleotide sequence (*p_NL_* = 45) with the first A-site nucleotide at position *p_NA_* = 22 (Supplementary Figure S1).

**Figure 5.**
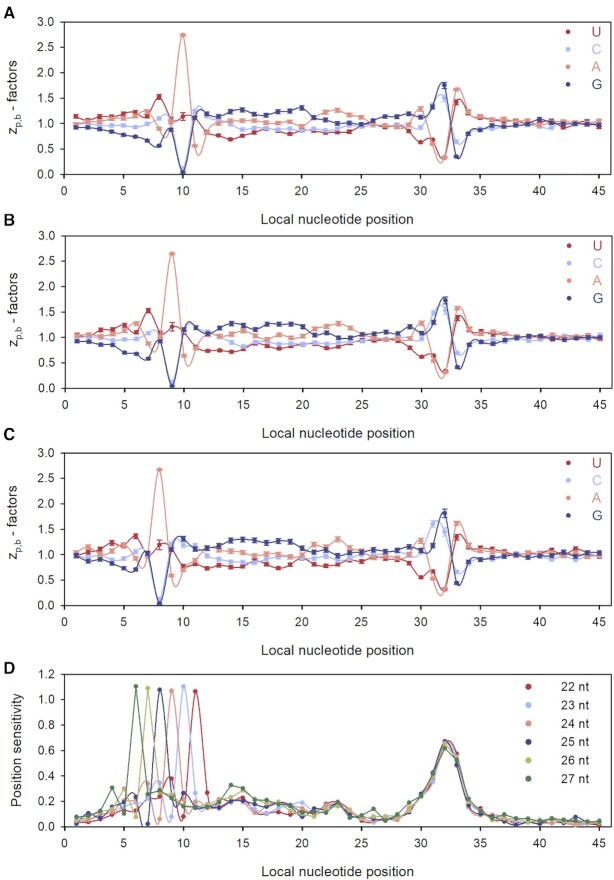
Context factors, $z_{p.b}^{FL}$ displayed for local nucleotide positions 1 to 45 for: (**A**) FL = 23 nt, (**B**) FL = 24 nt, (**C**) FL = 25 nt; *p* = 22 corresponds to the first A-site position (Supplementary Figure S1). (**D**) Position sensitivity profiles for $z_{p.b}^{FL}$-parameters calculated from RPF genome coverage with RPF fragments of lengths ranging from 22 to 27 nts.

We used our maximum likelihood (ML) approach to estimate the local context factors $z_{p.b}^{FL}$ that estimate the nucleotide context dependent variation in $\lambda _{ij}^{FL}$ as modelled by $c_{ij}^{\bmod ,FL}$ using Equations 19 and 20. Those $z_{p.b}^{FL}$ factors calculated for fragment length-specific experimental coverage profiles, $c_{i,j}^{\exp ,FL}$, are shown in Figure 5 for FL = 23, 24 and 25 nt. $z_{p.b}^{FL}$ varied greatly in response to changing nucleotide base identity (*b*) at positions 10, 9 and 8 for FL = 23 (Figure 5A), FL = 24 (Figure 5B) and FL = 25 nt (Figure 5C), respectively. At these combinations of nucleotide positions and lengths the $z_{p.b}^{FL}$ factors were always relatively small when *b* = G or *b* = C leading to small model (‘mod’) $g_{ij}^{\bmod ,FL}$ and $c_{ij}^{\bmod ,FL}$ values (Equations 19 and 20). Local positions *p* equal to 10, 9 and 8 correspond to 5′- ends of the FL = 23, 24 or 25 nts fragments, respectively, implying low abundance of RPFs with G/C at their 5′-ends. Indeed, experimental RPFs with an A at their 5'- end are about 60-fold more abundant than experimental RPFs with a G at the 5′- end, in line with the previous report on strong preference of MNase to cleave before an A or a U (9). Notably, the 5′-peak of the position sensitivity to nucleotide identity (calculated analogously to *S*_p_ in Equation (18)) moves exactly one nucleotide to the right as the fragment length increases by one nucleotide from 22 to 27 nt (Figure 5D). Irrespective of fragment length, the 3′-ends of RPFs are always aligned at local position *p* = 32, so that MNase cleavage occurs between positions 32 and 33 in the local nucleotide context (Figure 5). The $z_{p.b}^{FL}$ parameters with G or C at local position *p* = 33 were much smaller and those with A or U much larger than 1 (Figure 5), also in line with the observation that MNase cleaves before A/ U nts (9). The 3′-end cleavage bias of MNase was strong and yet considerably less pronounced than the 5′-end cleavage bias. In the local nucleotide region between positions 13 and 29 well inside the ribosome (Supplementary Figure S1), the $z_{p.b}^{FL}$ factors were very similar for different fragment lengths (Figure 5), suggesting insignificant technical bias in the 13–29 region of the local nucleotide context.

$z_{p,b}^{}$ factors estimated from the standard experimental RPF coverage profile, $c_{i,j}^{\exp }$, obtained by summation of length-specific experimental RPF coverage profiles $c_{i,j}^{\exp ,FL}$ for RPF lengths from 22 to 27 nts, exhibit much reduced 5′-bias but essentially unchanged 3′-bias (Supplementary Figure S4). The great reduction of the 5′-bias is easily understood by considering that the summation of length specific RPF profiles $c_{i,j}^{\exp ,FL}$ corresponds roughly to an FL-averaging of $z_{p.b}^{FL}$ factors. This also explains why the position sensitivity profile of $z_{p.c}^{}$ factors at codon resolution (Figure 4B) has a smaller bias at positions close to the lagging than to the leading edge of the ribosome.

The strong effects of codon identities at position 11 (leading edge of the ribosome) on ${z_{11,c}}$ values (Figure 4A) can now be easily explained by the biases at positions 31, 32 and 33 observed at nucleotide resolution. For example, the combinations of G or C at positions 31 and 32 with A or U at position 33 (corresponding to the three nucleotide positions of codon 11) are expected to result in large ${z_{11,c}}$-values, while U or A at positions 31 and 32 combined with C or G at position 33 should result in small ${z_{11,c}}$-values (see Figure 5 or Supplementary Figure S4). Indeed, GGU (Gly) and CCA (Pro) codons have ${z_{11,c}}$-values much larger than 1, while codons UUC (Phe) and AAG (Lys) have ${z_{11,c}}$-values much smaller than 1 (Figure 4A), exactly as predicted from 3′ biases (Supplementary Figure S4). The same analysis applied to the 5′ biases (Supplementary Figure S4) explains the strong ${z_{p,c}}$ codon dependence at the ribosomal lagging edge positions 3 and 4 (Figure 4).

**Figure 6.**
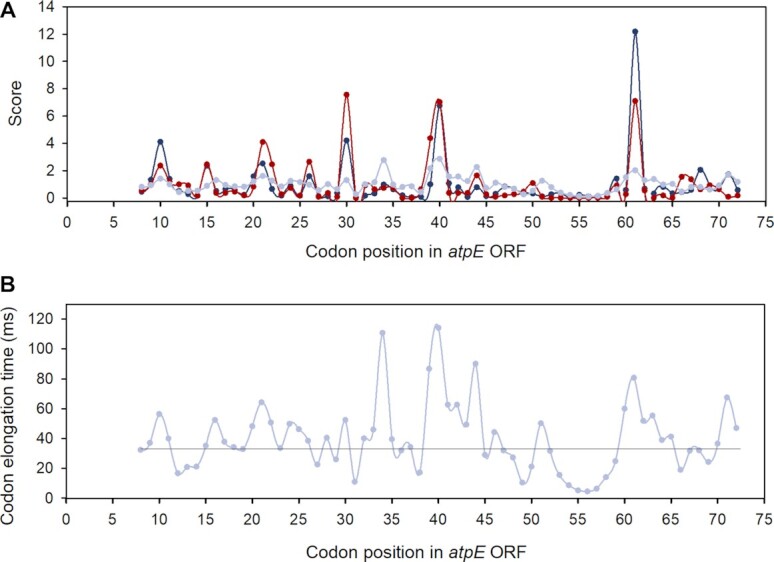
Pausing score profile and absolute time spectrum for the *atpE* (ATP synthase subunit C) transcript at single codon resolution. (**A**) Comparison of an experimental RPF score profile $s_{ij}^{\exp }$ (red, Equation 12) with the total model RPF score profile $s_{ij}^{\bmod }$ (blue, Equation 13) and model pausing score profile $s_{ij}^T$ (light blue, Equation 27). The pausing score profile is much less jagged ( $\sigma _i^T$ = 0.3) than the total model ($\sigma _i^{\bmod }$ = 0.9) and experimental ($\sigma _i^{\exp }$ = 1.1) RPF score profiles. (**B**) Absolute elongation time spectrum $t_{ij}^{\bmod }$ (Equation 39); the horizontal line corresponds to the average per-codon translation time of the *atpE* transcript.

For the codon resolution data, we conclude that the outer codon context-dependent ${z_{p,c}}$ factors for positions *p* = 1 to *p_1_–1* and from *p_2_+1* to *p_L_* (Figure 1) account for the technical biases in RPF library generation. In contrast, the inner codon context ${z_{p,c}}$ factors, for positions *p* from *p_1_* to *p_2_* mainly reflect the context dependent variation of the peptide elongation times. With this as a lead we estimated the ${z_{p,c}}$ factors for the *E. coli* AS19 dataset and used the inner subset of ${z_{p,c}}$ factors for all positions from *p_1_* = 5 to *p_2_* = 9 to obtain bias-corrected model $g_{ij}^T$ parameters (Equation 25). A typical example of such bias elimination is shown in Figure 6A for the *E. coli atpE* transcript. We contend that the bias-corrected model $s_{ij}^T$ pausing scores (Equation 27) reflect the bias-free peptide elongation times showing that the ribosome translates mRNA in a much smoother fashion than the experimental $s_{ij}^{\exp }$ RPF scores might suggest.

We have also estimated the absolute peptide elongation time, $\tau _{ij}^{} = {\tau ^e}\gamma _{ij}^T$, as the product $t_{ij}^{\bmod } = {t^e}g_{ij}^T$ where ${t^e}$ estimates the time factor ${\tau ^e}$ in Equation 1 (Figure 6B). We note that our modeling approach allows for determination of the model $g_{ij}^T$ parameters, and, hence, model pausing scores $s_{ij}^T$ from the ribosome profiling data alone, but for ${t^e}$ calculation we need to use additional experimental information provided by the growth rate $\mu$ of the bacterial population (Equation 38).

### The local codon context dependent distribution of relative peptide elongation times

The elimination of the technical bias described in the previous section enables estimation of authentic peptide elongation times for any A-site codon *j* in any ORF*_i_* by ‘dividing out’ the bias dependent local context parameter $g_{ij}^B$ (Equation 24) from the total context parameter $g_{ij}^{\bmod }$ (Equation 6), which leads to the context parameter $g_{ij}^T$ (Equation 25) proportional to the A-site codon elongation time $t_{ij}^{\bmod }$ (Equation 30). The frequency densities of $g_{ij}^{\bmod }$ and bias-free $g_{ij}^T$ values for the *E. coli* transcriptome are displayed along with those for their logarithms in Supplementary Figure S5. The frequency densities of $g_{ij}^T$ and $g_{ij}^{\bmod }$ logarithms are near Gaussian with σ-values of 0.61 and 1.2, respectively (Supplementary Figure S5B). From this, we propose that each rate-limiting elongation step involves the passage over a standard free energy barrier determined by the sum of standard free energy contributions determined by the logarithms of ${z_{p,c}}$ factors in the local codon context. According to the transition-state theory, the time it takes to overcome a standard free-energy barrier increases exponentially with the barrier height (31). In translocation, the height of the free energy barrier could be the sum of the free energies of interaction between ribosome and mRNA throughout the whole inner context region. In peptidyl transfer, the barrier height could be the sum of the free energies from the identities of codons upstream of the A-site codon. According to the Central Limit Theorem, the frequency densities of such free energy sums would be near-Gaussian, providing a tentative explanation for the near-Gaussian frequency densities of the logarithm of $g_{ij}^T$-values (Supplementary Figure S5B) the exponentiation of which then leads to a log-normal distribution (Supplementary Figure S5A). Interestingly, frequency density of a log-normal distribution is mimicked by the distribution of the sum of two stochastic variables, one normally and one exponentially distributed. Possibly, this feature has led to the previous proposal that there are two-time components in peptide elongation, one Gaussian and one exponential (32). Finally, we note that due to the local context dependent bias there are more $z_{p,c}^{}$ factors in $g_{ij}^{\bmod }$ (Equation 6) than in $g_{ij}^T$ (Equation 25), leading to a broader near-Gaussian distribution for the logarithm of $g_{ij}^{\bmod }$ than of $g_{ij}^T$ (Supplementary Figure S5B).

### Determinants of fast and slow peptide elongation cycles in *E. coli*

**Figure 7.**
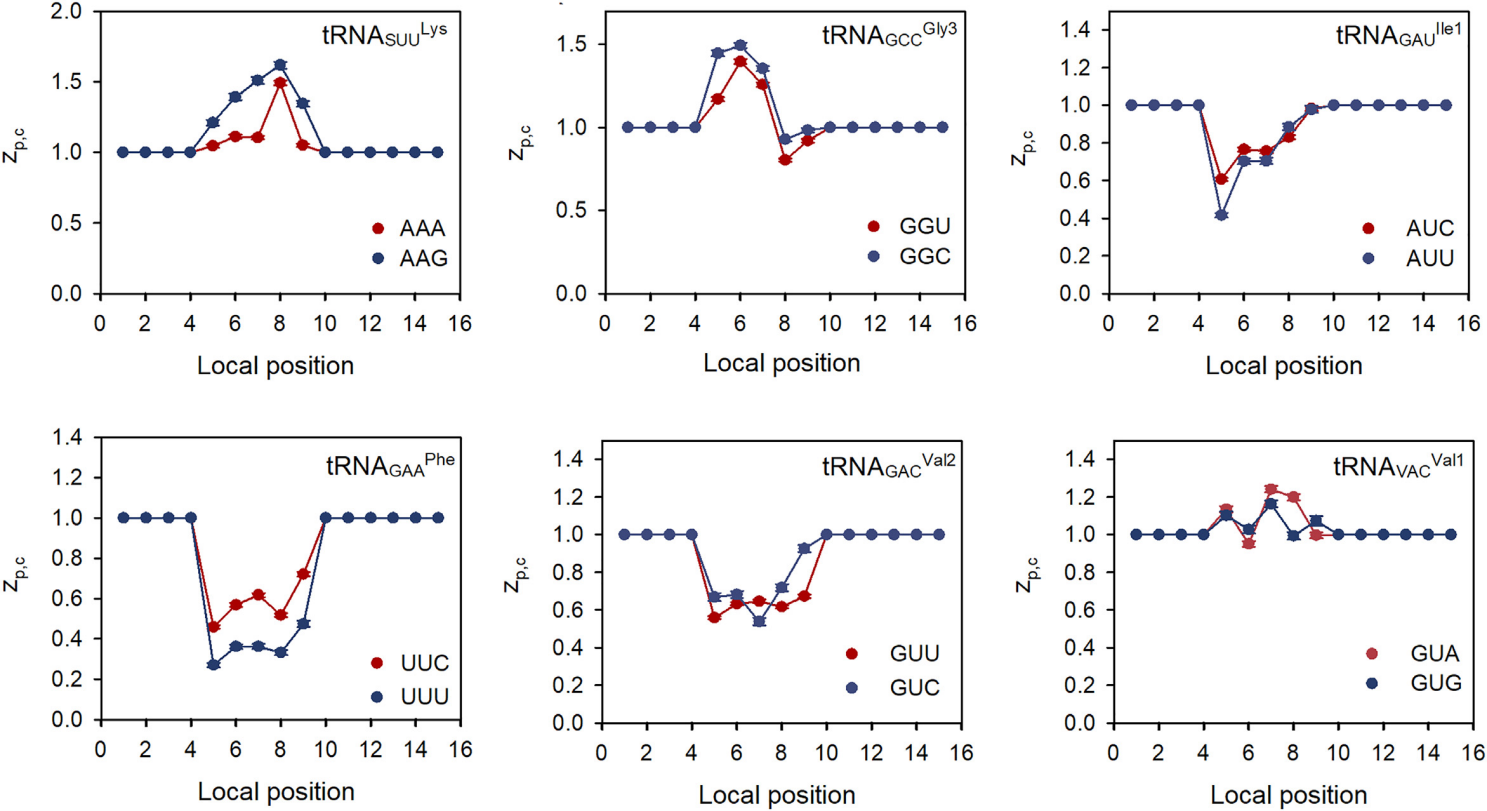
Variation of z-factors with the local codon position around the A site (*p*_A_= 8) for selected tRNAs reading synonymous codons. Large and small ${z_{p,c}}$-values contribute to slow and fast peptide elongation, respectively. A complete set of *z*-factors for all tRNAs is presented in Supplementary Figure S6.

The model estimate $t_{ij}^{\bmod }$ (Equation 30) of the time that the ribosome spends translating codon *j* of ORF*_i_* is proportional to $g_{ij}^T$ (Equation 25), a parameter which is estimated from product of $z_{p,c}^{}$ factors for the inner codons of the local context around the A site in the *p*-interval from 5 to 9 (Figure 1). Accordingly, the size of each inner ${z_{p,c}}$ factor is a determinant of the peptide elongation time. Under our experimental *E. coli* AS19 growth conditions the $z_{p,c}^{}$ values for Lys codons AAA or AAG pairing to tRNA^Lys^ in A (*p* = 8), P (*p* = 7) or E site (*p* = 6) were relatively large and contributed to slow peptide elongation (Figure 7). A similar picture holds for Gly codons GGU and GGC, read by tRNA^Gly3^. In contrast, Ile codons AUC and AUU, Phe codons UUU and UUC and Val codons GUC and GUU translated by tRNA^Ile2^, tRNA^Phe^ and tRNA^Val2^, respectively, exhibited relatively small ${z_{p,c}}$ values in the A, P and E site of the local context and contributed to fast peptide elongation (Figure 7). In most cases, synonymous codons read by the same tRNA isoacceptor have similar ${z_{p,c}}$ values (Figure 7 and Supplementary Figure S6). This, we propose, reflects similar interactions between the ribosome and the shared cognate tRNA. Along the same line, inner ${z_{p,c}}$ factors of Val codons at the same local position *p* were different when read by tRNA^Val2^ or tRNA^Val1^ (Figure 7), probably reflecting different interactions between the ribosome and the bodies of tRNA^Val2^ and tRNA^Val1^.

**Figure 8.**
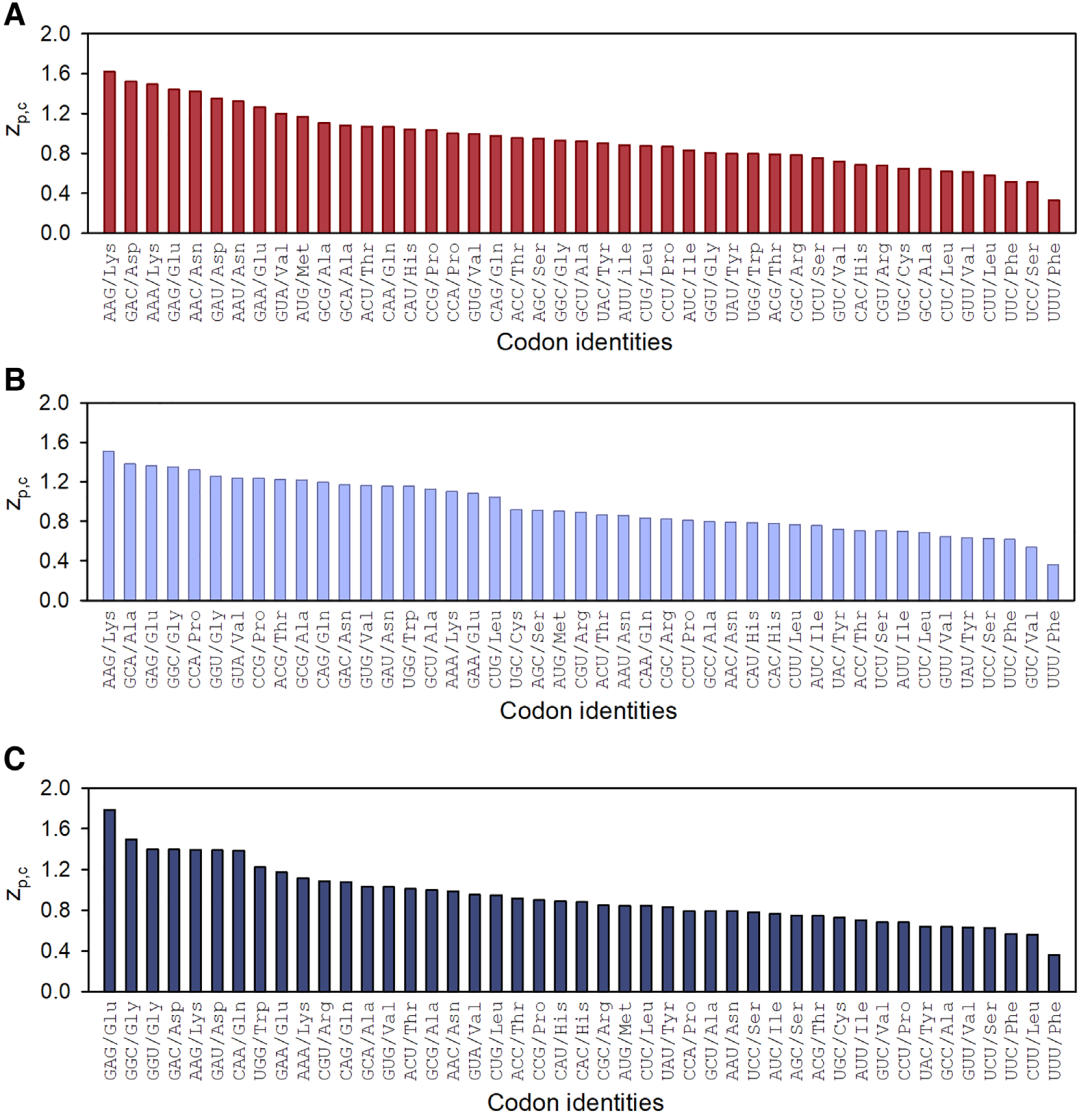
Codons ranked according to ${z_{p,c}}$ values for (**A**) A-site, (**B**) P-site and (**C**) E-site position of local context. Large and small ${z_{p,c}}$-values designate slow and fast peptide elongation, respectively. Codons are ordered in the descending order of ${z_{p,c}}$ values.

In the A site, codons for charged AAs, e.g. Lys, Asp and Glu, and one hydrophobic AA, Val, encoded by the GUA codon promoted slow peptide elongation (Figure 8A). Codons encoding Gly, Pro and Ala promoted fast or slow peptide elongation depending on whether they are in the A or P site of the local context (Figure 8A, B). In the E site of the local context codons encoding Lys, Glu, Gln and Asp as well as the Gly codons GGC and GGU (translated by tRNA^Gly3^) contributed to slow peptide elongation (Figure 8C). Codons encoding aromatic AAs generally promoted fast elongation when in A, P and E sites of the local context, with Phe being the fastest for our dataset.

### Peptide elongation times in conditions of ternary complex depletion

**Figure 9.**
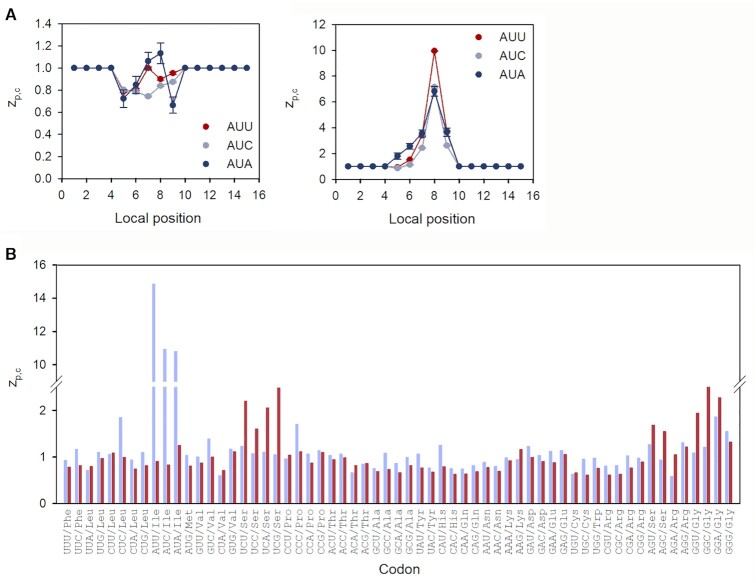
${z_{p,c}}$ values are affected by *E. coli* MG 1655 amino acid starvation. (**A**) Marked stalling at Ile codons following treatment with mupirocin. Variation of ${z_{p,c}}$ factors with the local codon position around the A site (*p*_A_= 8) for three Ile codons in untreated *E. coli* MG1655 (left panel) and treated with mupirocin (right panel). Large ${z_{p,c}}$ values indicate propensity for slow peptide elongation. (**B**) Comparison of the A-site ${z_{p,c}}$ values for untreated *E. coli* MG 1655 (red) and *E. coli* MG 1655 treated with mupirocin (light blue). Codons are ordered as in the genetic code table.

Next, we considered two published Ribo-Seq datasets, one generated from *E. coli* MG 1655 strain following short incubation with mupirocin and the other representing an untreated control, grown under otherwise identical conditions (6). Mupirocin is an inhibitor of isoleucyl-tRNA synthetase (IleRS) (33), which depletes charged tRNA^Ile^ and causes strong A-site pausing at Ile codons (6). Accordingly, our analysis of the dataset with mupirocin treatment showed greatly increased ${z_{p,c}}$ values for all three Ile codons at A site which correlated with slow peptide elongation at Ile codons due to reduced supply of Ile-tRNA^Ile^-containing ternary complexes (Figure 9A). We noted also that for the major Ile codons (AUC and AUU) ${z_{p,c}}$ increased by 13- and 16-fold, respectively, whereas ${z_{p,c}}$ for the minor AUA Ile codon increased 8.5-fold (Figure 9A). Since the concentration of the minor AUA reading tRNA^Ile2^ is an order of magnitude lower than the tRNA^Ile1^ concentration pairing to the major Ile codon (34), we propose that the mupirocin-induced relative increase in A-site binding time is much larger for ternary complex with the major than with the minor isoacceptor (see Discussion for more details). A much higher sensitivity to IleRS inhibition for AUC/AUU than for AUA reading is also predicted by the theory of selective charging of tRNA isoacceptors (35), corroborated for a similar case of other aminoacyl-tRNA synthetase inhibition (36).

Compared to the untreated *E. coli* MG1655 cells we also found that along with the slower Ile codon reading, mupirocin addition caused a faster Ser/Gly codon decoding (Figure 9B). The relatively slow decoding of Ser and Gly codons in the control was attributed to quick depletion of Ser- and Gly-tRNAs due to culture filtration before the Ribo-Seq library preparation (6). Accordingly, we also detected higher ${z_{p,c}}$ values at Ser and Gly codons indicative of slow elongation on these codons in the untreated *E. coli* MG1655 cells (Figure 9B). We speculate that mupirocin treatment results in a drastic slowing down of the global translation in the cell, which also reduces Ser and Gly consumption. Hence, the pools of charged seryl-tRNAs and glycyl-tRNAs are maintained, thus eliminating the pausing on Gly and Ser codons (see Discussion for details).

We have also calculated $z_{p.b}^{FL}$ factors at nucleotide resolution for the untreated *E. coli* MG 1655 data set (Supplementary Figure S7A) and compared them with $z_{p.b}^{FL}$ factors for our dataset (Figure 5). While the 3′-end bias in $z_{p.b}^{FL}$ factors for the same FL was similar for the two data sets, the 5′-end bias was much less pronounced for the *E. coli* MG 1655 (compare Figure 5A and S7A). Similarly, ${z_{p,b}}$ factors estimated from the standard RPF nucleotide coverage profile $c_{i,j}^{\exp }$ for *E. coli* MG 1655 also had much less pronounced 5′- bias than the corresponding $z_{p.b}^{}$ factors for the *E. coli* AS19 data set (Supplementary Figures S7B and S4). We attribute these differences to the much longer incubation with MNase of 1 hour for *E. coli* MG1655 (6) vs. 10 min for our *E. coli* AS19 during library preparations.

### Neutralization of nuclease induced bias in Ribo-seq spectra from *Saccharomyces cerevisiae*

**Figure 10.**
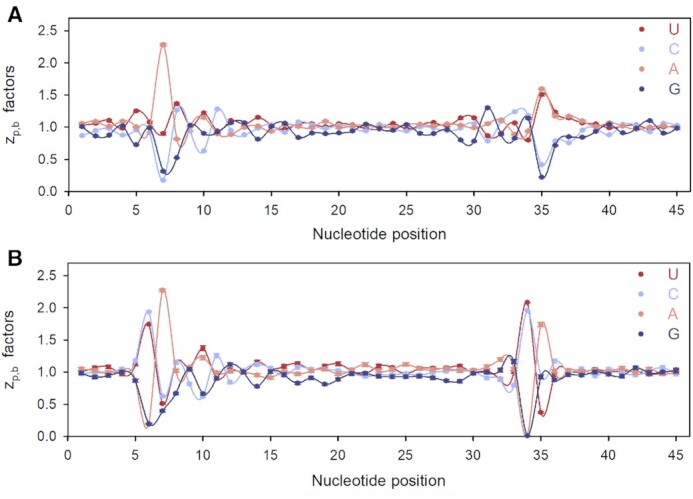
Nucleotide-resolution $z_{p.b}^{FL}$-factors calculated from RPF coverage profiles with RPFs with FL = 28 for yeast Ribo-Seq datasets constructed using MNase (**A**) and RNase A (**B**).

To further validate our modeling approach, we considered two published datasets from the yeast *S. cerevisiae* (19). These were prepared with MNase (S7) and RNase A with distinct cleavage biases: while MNase cuts preferentially before A and U, RNase A cleaves preferentially after C and U (19). Here, we applied our nucleotide-resolution ML approach to quantify the characteristic biases in the two datasets. For the MNase set we detected much higher $z_{p.b}^{FL}$ factor values for A/U compared to C/G nucleotides at position *p* = 7 and *p* = 35 corresponding to the nucleotides at the 5′- end and the nucleotide after the 3′- RPF end, respectively (Figure 10A). This pattern is very similar to that in our MNase dataset from *E. coli* (Figure 5). In contrast, the $z_{p.b}^{FL}$ factors for the RNaseA dataset were relatively small for *b* = A or G at position *p* = 6, corresponding to the nucleotide before the 5′- end of the RPF and near zero at position *p* = 34 which corresponds its 3′- end (Figure 10 B). This suggests that the technical biases of the two data sets are distinct and the differences reflect the cleavage preferences of the nucleases used to generate the RPF libraries.

**Figure 11.**
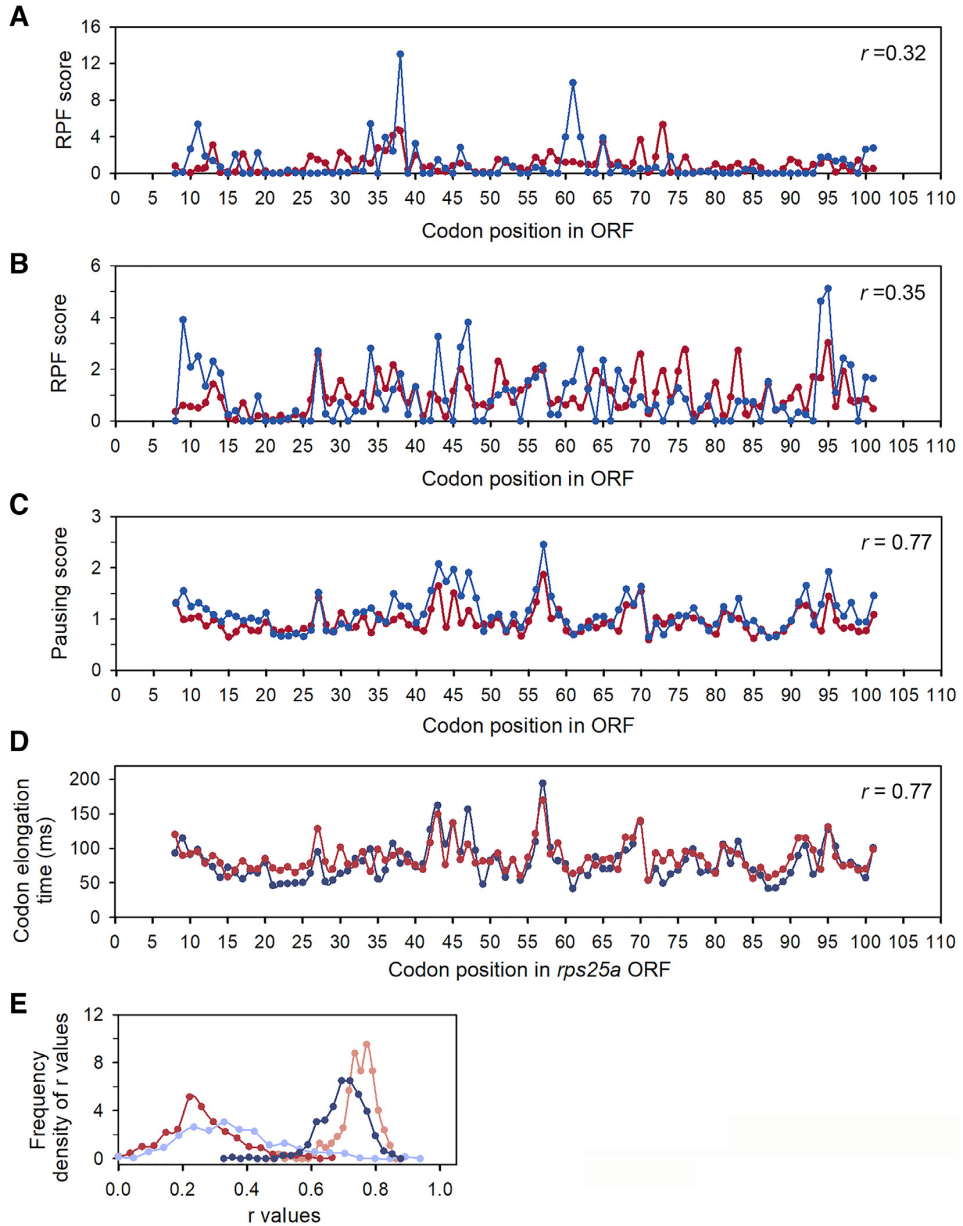
Comparison of experimental RPF scores $s_{ij}^{\exp }$ (**A**), model RPF scores $s_{ij}^{\bmod }$ (**B**) and pausing scores $s_{ij}^T$ (**C**) for YGR027C transcript (encoding ribosomal protein S25, Rps25a) in MNase (red) and RNase A (blue) yeast Ribo-Seq data sets; the Pearson correlation coefficients, *r*, between the two data sets are *r* = 0.32 (A), *r* = 0.35 (B), *r* = 0.77 (C) and *r* = 0.77 (D). (**D**) Absolute elongation time spectrum (Equation 39) of YGR027C derived from the MNase (red) and RNase A (blue) Ribo-Seq data set. The mean elongation time per codon for the YGR027C transcript is 80.8 ms and 86.7 ms when estimated from the RNAse A and MNase datasets, respectively. (**E**) Frequency distribution of Pearson correlation coefficients, *r*, between scores for the same transcripts in yeast datasets prepared with RNase A (RA) and MNase (S7): for experimental scores (light blue, correlation between $s_{ij}^{\exp ,RA}$ and $s_{ij}^{\exp ,S7}$), model scores (red, correlation between $s_{ij}^{\bmod ,RA}$ and $s_{ij}^{\bmod ,S7}$), ‘five-inner model’ scores (dark blue, correlation between $\tilde{s}_{ij}^{\bmod ,RA}$ and $\tilde{s}_{ij}^{\bmod ,S7}$) and bias-free pausing scores (orange, correlation between $s_{ij}^{T,RA}$ and $s_{ij}^{T,S7}$). ‘Five-inner model’ refers to modeling each (*i*,*j*) A-site context contribution with 5 $\tilde{z}_{p,c}^{}$ position parameters (see Equations 28 and 29 for $\tilde{s}_{ij}^{\bmod }$ definition).

We then calculated the codon resolution ${z_{p,c}}$ factors for the MNase- and RNase A-treated yeast datasets and used the inner subset of ${z_{p,c}}$ factors for positions from *p_1_* = 5 to *p_2_* = 9 to obtain bias-corrected $g_{ij}^T$ parameters (Equation 25) and $s_{ij}^T$ pausing scores (Equation 27). As expected, both the correlation between the $s_{ij}^{\exp }$ RPF scores (Equation 12) and between the $s_{ij}^{\bmod }$ RPF scores (Equation 13) from the two data sets obtained with MNase and RNase are weak (*r* = 0.3) as exemplified in Figure 11A and B for the YGR027C transcript (coding for the S25 protein of the 40S ribosomal subunit). In contrast, the bias-corrected pausing score $s_{ij}^T$ profiles of YGR027C from the two data sets are strongly correlated (*r* = 0.77) with similar features (Figure 11C). The absolute translation time profiles $t_{ij}^{\bmod }$ for YGR027C transcript calculated from RNase A and MNase data sets, assuming 2 h duplication time of the yeast culture (Equation 36), are also remarkably similar (Figure 11D). This similarity also reflects the varying codon elongation time as the ribosome moves codon by codon along the YGR027C transcript. We obtained very similar results for other transcripts. Notably, the frequency distribution of the *r* -values underwent a large shift from low to high correlation following neutralization of nuclease-introduced biases (Figure 11E). We also observed a strong correlation between $z_{p,c}^{S7}$ and $z_{p,c}^{RA}$ values obtained for the MNase (S7) and RNase A datasets for positions near the A site (Supplementary Figure S8A). This correlation for the A-site position was *r* = 0.8 and increased (*r* = 0.85) when rear codons (i.e. with frequency < 0.3%) were excluded. This, we suggest, reflects the similarity of the effects of a particular A-site codon on the codon translation time in both data sets. For P and E sites, the correlation between $z_{p,c}^{S7}$ and $z_{p,c}^{RA}$ factors for the P and E sites was less pronounced (*r* = 0.7). As expected, the correlation between $z_{p,c}^{S7}$ and $z_{p,c}^{RA}$ factors for positions near the edges of the yeast ribosome (e.g. positions 3 and 12) is low (*r* < 0.25), which reflects the distinct sequence preferences of the nucleases in RPF generation (Supplementary Figure S8B).

We wish to emphasize that to obtain the bias-corrected pausing scores $s_{ij}^T$ (Equation 27), we used the five inner-position ${z_{p,c}}$ values from the fifteen ${z_{p,c}}$ values accounting for the total local A-site context in the modeling of the experimental $c_{ij}^{\exp }$ datasets. Different bias elimination method has been developed earlier (20) using neural network modeling to predict the elongation time of the A-site codon from its short sequence context (that does not include the edges of the RPFs). To compare these two principally different ways of bias elimination, in similarity to this approach (20), we restricted the context to five codons around each A-site codon, and, hence excluded the edges of the RPFs. We then modeled the two Ribo-Seq sets from *S. cerevisiae* (19) processed with either MNase or RNase A using “five-inner modeling", i.e. using only five-inner $\tilde{z}_{p,c}^{}$ values to obtain RPF scores $\tilde{s}_{ij}^{\bmod }$ (Equations 28 and 29) and found them different from pausing scores $s_{ij}^T$. We then calculated Pearson correlations between $\tilde{s}_{ij}^{\bmod ,RA}$ and $\tilde{s}_{ij}^{\bmod ,S7}$ for each transcript *i* in the two datasets. Clearly, our ‘bias-free method’ leads to much higher correlation between the ${z_{p,c}}$ derived $s_{ij}^T$ scores (Figure 11E, orange ‘bias-free’ *r*-frequency profile) than the ‘five-inner model’ for the correlation between the ${\tilde{z}_{p,c}}$-derived $\tilde{s}_{ij}^{\bmod }$ scores (Figure 11E, ‘five inner model’, dark blue *r*-frequency profile). An intuitive explanation for this result is that in the ‘five inner model’ the five $\tilde{z}_{p,c}^{}$ inner-position factors used for the description of a $c_{ij}^{\exp }$ dataset absorb the experimental biases. In contrast, using *p_L_* = 15 local positions for modeling $c_{ij}^{\exp }$, the experimental biases are absorbed by the outer ten ${z_{p,c}}$ factors (Equation 24), thus leaving the five-inner ${z_{p,c}}$ factors bias-free. Thus, the ‘five-inner modelling’ that essentially emulates an earlier approach (20), reduces the precision of elongation time estimates.

## DISCUSSION

Since decades, quantitative studies of protein synthesis with purified ribosomes and auxiliary translation components have been performed across species (37,38). In spite of the insights from these biochemical approaches, there are considerable differences between the empirical contexts of cell-free and intracellular mRNA translation. For instance, in the living cell tightly controlled parallel pathways exist for the supply of aminoacyl-tRNAs, for ternary complex formation and, furthermore, the translation of A-site codons takes place in the context of a virtual infinitude of sets of neighboring codons. Thus, experimental approaches orthogonal to *in vitro* biochemistry will deepen our understanding of how the intracellular kinetic networks of mRNA translation shape the life sustaining phenotypes of living cells. In the present work, we join the ongoing and rapidly growing efforts to establish genome-wide technologies (3,39) for quantitative studies of mRNA translation. We provide a framework for parallel estimation of elongation times of all codons in all local codon contexts of different types of cells. This was made possible by the development of novel type of model to be fitted to transcriptome-wide ribosome profiling data for parameters estimation. Our model describes the elongation time at each codon of the transcriptome as a product of 15 independent ${z_{p,c}}$ factors, one for each codon position in the local context surrounding the ribosomal A site. The factor for each codon context position can have one of 61 possible values, depending on its codon identity and context position. Using a maximum likelihood criterion, we obtain the values of 15 × 61 = 915 ${z_{p,c}}$ factors for 61 sense codons in 15 local context positions by fitting our model to the experimental RPF spectrum. Despite large ruggedness and stochastic fluctuations, the experimental data are well fitted by the model.

To discriminate between effects of codon context on nuclease cleavage preferences on one hand and peptide elongation time variations on the other, we use models with both single-codon and single-nucleotide resolution. In line with previous findings (9), we find much higher MNase activity at A/U compared to G/C nucleotides near the 5′- or 3′-ends of RPFs, leading to strongly skewed fragment creation/processing and biased RPF spectra. At the same time, the MNase cleavage bias does not propagate into the inner context on both sides of the A-site codon, a crucial feature enabling neutralization of technical codon context-dependent bias. In this way, we derived unbiased RPF spectra suitable for estimation of codon elongation times throughout the transcriptome. We observed differences in the ${z_{p,c}}$ values for the A site between two different *E. coli* strain, MG1655 and AS19 (compare Figures 8A and 9B), implying that our approach can very sensitively detect elongation time difference at single codon between different strains, growth medium and conditions.

We have applied our modeling approach to clarify the effects of mupirocin-induced inhibition of the IleRS activity in a bacterial system using a previously published data set (6). The inhibition decreases the rate of supply of charged tRNA^Ile^ isoacceptors (33) and greatly enhances values of ${z_{p,c}}$ parameters for all three Ile codons (AUA, AUC or AUU) in the A site, suggesting greatly increased binding time for isoleucyl-tRNA^Ile^-containing ternary complexes. Considering that the total concentration of major tRNA^Ile1^ isoacceptor is an order of magnitude larger than that of the minor tRNA^Ile2^ isoacceptor (34) and assuming nearly 100% charged levels of both tRNA^Ile^ isoacceptors in the absence of the inhibitor, the time for ternary complex binding into the A site is estimated to be an order of magnitude smaller for AUC/AUU than for AUA codons. In the inhibitor-less case, the total peptide elongation time is about 30% longer for AUA than for AUC/AUU codons (Figure 9B). From these data we suggest that the relative change in the time for ternary complex binding into the A site is much larger for AUC/AUU than AUA codons, meaning that AUA decoding is much less sensitive to IleRS inhibition than AUC/AUU. This further corroborates the theory of selective charging of tRNA isoacceptors (35), previously validated by SerRS inhibition in *E. coli* cells (36). In fact, our method might be very useful for detection of ternary complex depletion scenarios in cells. This optimistic notion receives further support from the observation that mupirocin, in addition to slowing down translation at Ile codons, also speeds up translation of Gly and Ser codons in a codon selective manner. That is, mupirocin addition reduces considerably the reading times of major (GGC/GGU) but not of the minor (GGG/GGA) Gly codons and reduces the reading times for all Ser codons (Figure 9). A possible scenario to explain also these codon-specific patterns is that under experimental conditions used to obtain the RPF dataset in *E. coli* MG1655 grown in balanced medium both Gly and Ser codons are weakly starved for their cognate ternary complex (6) due to deficient intracellular supply of Gly and Ser (40). Mupirocin addition slows down the overall protein synthesis, thereby removing the supply bottlenecks of Gly and Ser and pausing at their codons. We note that the theory of selective charging of tRNAs predicts starvation-sensitive reading of GGC/GGU but not of GGG/GGA codons and starvation sensitivity of all Ser codons (35), which corroborates the proposed scenario of weak Gly and Ser starvation that is removed by addition of an IleRS inhibitor.

We have broadened our approach from bacterial systems to include also eukaryote systems. We compared two published Ribo-Seq sets from *S. cerevisiae* (19), derived from identical yeast populations but processed with different nucleases, either MNase or RNase A. Both RNases exhibit strong but distinct cleavage preferences leading to greatly different and virtually uncorrelated experimental and model reproduced RPF spectra. However, after bias neutralization model spectra for both RNases become less rugged and are strikingly similar (Figure 11). This means, we propose, that our bias-neutralization approach provides a solution to the long-standing problem of extracting reliable quantitative information about individual codon elongation cycle times from greatly rugged, highly noisy and biased RPF spectra.

Ribosome profiling holds a great promise of detailed insights into the dynamics of protein synthesis in single cells and multicellular organisms. The ongoing improvements of data analysis along with refinements of experimental techniques and the synergy of different and sometimes orthogonal approaches will accelerate the development of this promising field.

## DATA AVAILABILITY

The sequencing data for *E. coli* AS19 generated in this study have been deposited within Gene Expression Omnibus (GEO) under accession number GSE145571. Two published data sets (6,19) analyzed here too, are available under the accession numbers in the GEO Series with accession number GSE119104 (GSM3358136 and GSM3358137) for *E. coli* MG1655 and GSE 82220 (GSM2186726 and GSM2186728) for yeast. All scripts and source code for modeling and calculating the parameters used here are deposited in https://github.com/gustafGitHub/RiboTimes.

## Supplementary Material

gkab260_Supplemental_File

## References

[1] Steitz J.A. Polypeptide chain initiation: nucleotide sequences of the three ribosomal binding sites in bacteriophage R17 RNA. Nature. 1969; 224:957–964. 5360547 10.1038/224957a0

[2] Ingolia N.T. , Ghaemmaghami S., Newman J.R., Weissman J.S. Genome-wide analysis in vivo of translation with nucleotide resolution using ribosome profiling. Science. 2009; 324:218–223. 19213877 10.1126/science.1168978 PMC2746483

[3] Ingolia N.T. Ribosome footprint profiling of translation throughout the genome. Cell. 2016; 165:22–33. 27015305 10.1016/j.cell.2016.02.066 PMC4917602

[4] Schuller A.P. , Green R. Roadblocks and resolutions in eukaryotic translation. Nat. Rev. Mol. Cell Biol. 2018; 19:526–541. 29760421 10.1038/s41580-018-0011-4 PMC6054806

[5] Stern-Ginossar N. , Ingolia N.T. Ribosome profiling as a tool to decipher viral complexity. Annu Rev Virol. 2015; 2:335–349. 26958919 10.1146/annurev-virology-100114-054854

[6] Mohammad F. , Green R., Buskirk A.R. A systematically-revised ribosome profiling method for bacteria reveals pauses at single-codon resolution. eLife. 2019; 8:e42591. 30724162 10.7554/eLife.42591 PMC6377232

[7] Woolstenhulme C.J. , Guydosh N.R., Green R., Buskirk A.R. High-precision analysis of translational pausing by ribosome profiling in bacteria lacking EFP. Cell Rep. 2015; 11:13–21. 25843707 10.1016/j.celrep.2015.03.014 PMC4835038

[8] Datta A.K. , Burma D.P. Association of ribonuclease I with ribosomes and their subunits. J. Biol. Chem. 1972; 247:6795–6801. 4563069

[9] Dingwall C. , Lomonossoff G.P., Laskey R.A. High sequence specificity of micrococcal nuclease. Nucl Acis Res. 1981; 9:2659–2673. 10.1093/nar/9.12.2659 PMC326883 6269057

[10] O’Connor P.B. , Li G.W., Weissman J.S., Atkins J.F., Baranov P.V. rRNA:mRNA pairing alters the length and the symmetry of mRNA-protected fragments in ribosome profiling experiments. Bioinformatics. 2013; 29:1488–1491. 23603333 10.1093/bioinformatics/btt184 PMC3673220

[11] Weinberg D.E. , Shah P., Eichhorn S.W., Hussmann J.A., Plotkin J.B., Bartel D.P. Improved ribosome-footprint and mRNA measurements provide insights into dynamics and regulation of yeast translation. Cell Rep. 2016; 14:1787–1799. 26876183 10.1016/j.celrep.2016.01.043 PMC4767672

[12] Zheng W. , Chung L.M., Zhao H. Bias detection and correction in RNA-sequencing data. BMC Bioinfomat. 2011; 12:290. 10.1186/1471-2105-12-290 PMC3149584 21771300

[13] Artieri C.G. , Fraser H.B. Accounting for biases in riboprofiling data indicates a major role for proline in stalling translation. Genome Res. 2014; 24:2011–2021. 25294246 10.1101/gr.175893.114 PMC4248317

[14] O’Connor P.B. , Andreev D.E., Baranov P.V. Comparative survey of the relative impact of mRNA features on local ribosome profiling read density. Nat. Commun. 2016; 7:12915. 27698342 10.1038/ncomms12915 PMC5059445

[15] Sharma A.K. , Sormanni P., Ahmed N., Ciryam P., Friedrich U.A., Kramer G., O’Brien E.P. A chemical kinetic basis for measuring translation initiation and elongation rates from ribosome profiling data. PLoS Comput. Biol. 2019; 15:e1007070. 31120880 10.1371/journal.pcbi.1007070 PMC6559674

[16] Del Campo C. , Bartholomaus A., Fedyunin I., Ignatova Z. Secondary structure across the bacterial transcriptome reveals versatile roles in mRNA regulation and function. PLoS Genet. 2015; 11:e1005613. 26495981 10.1371/journal.pgen.1005613 PMC4619774

[17] Mortazavi A. , Williams B.A., McCue K., Schaeffer L., Wold B. Mapping and quantifying mammalian transcriptomes by RNA-Seq. Nat Meth. 2008; 5:621–628. 10.1038/nmeth.1226 PMC13303166 18516045

[18] Mohammad F. , Woolstenhulme C.J., Green R., Buskirk A.R. Clarifying the translational pausing landscape in bacteria by ribosome profiling. Cell Rep. 2016; 14:686–694. 26776510 10.1016/j.celrep.2015.12.073 PMC4835026

[19] Gerashchenko M.V. , Gladyshev V.N. Ribonuclease selection for ribosome profiling. Nucleic Acids Res. 2017; 45:e6. 27638886 10.1093/nar/gkw822 PMC5314788

[20] Tunney R. , McGlincy N.J., Graham M.E., Naddaf N., Pachter L., Lareau L.F. Accurate design of translational output by a neural network model of ribosome distribution. Nat. Struct. Mol. Biol. 2018; 25:577–582. 29967537 10.1038/s41594-018-0080-2 PMC6457438

[21] Levenberg K. A method for the solution of certain non-linear problems in least squares. Quarterly Appl Math. 1944; 2:164–168.

[22] Marquardt D. An algorithm for least-squares estimation of nonlinear parameters. SIAM J. Appl. Math. 1963; 11:431–441.

[23] Dana A. , Tuller T. The effect of tRNA levels on decoding times of mRNA codons. Nucleic Acids Res. 2014; 42:9171–9181. 25056313 10.1093/nar/gku646 PMC4132755

[24] Hussmann J.A. , Patchett S., Johnson A., Sawyer S., Press W.H. Understanding biases in ribosome profiling experiments reveals signatures of translation dynamics in yeast. PLoS Genet. 2015; 11:e1005732. 26656907 10.1371/journal.pgen.1005732 PMC4684354

[25] Bremer H. , Dennis P.P. Modulation of chemical composition and other parameters of the cell at different exponential growth rates. EcoSal Plus. 2008; 3:doi:10.1128/ecosal.5.2.3. 10.1128/ecosal.5.2.3 26443740

[26] Bartholomaus A. , Del Campo C., Ignatova Z. Mapping the non-standardized biases of ribosome profiling. Biol. Chem. 2016; 397:23–35. 26351919 10.1515/hsz-2015-0197

[27] McGlincy N.J. , Ingolia N.T. Transcriptome-wide measurement of translation by ribosome profiling. Methods. 2017; 126:112–129. 28579404 10.1016/j.ymeth.2017.05.028 PMC5582988

[28] Chiba S. , Ito K. Multisite ribosomal stalling: a unique mode of regulatory nascent chain action revealed for MifM. Mol. Cell. 2012; 47:863–872. 22864117 10.1016/j.molcel.2012.06.034

[29] Lu J. , Deutsch C. Electrostatics in the ribosomal tunnel modulate chain elongation rates. J. Mol. Biol. 2008; 384:73–86. 18822297 10.1016/j.jmb.2008.08.089 PMC2655213

[30] Nakatogawa H. , Ito K. Secretion monitor, SecM, undergoes self-translation arrest in the cytosol. Mol. Cell. 2001; 7:185–192. 11172723 10.1016/s1097-2765(01)00166-6

[31] Laidler K. , King C. Development of transition-state theory. J. Phys. Chem. 1983; 87:2657–2664.

[32] Dana A. , Tuller T. Properties and determinants of codon decoding time distributions. BMC Genomics. 2014; 15:S13. 10.1186/1471-2164-15-S6-S13 PMC4240079 25572668

[33] Hughes J. , Mellows G. Inhibition of isoleucyl-transfer ribonucleic acid synthetase in *Escherichia coli* by pseudomonic acid. Biochem. J. 1978; 176:305–318. 365175 10.1042/bj1760305 PMC1186229

[34] Ikemura T. Correlation between the abundance of Escherichia coli transfer RNAs and the occurrence of the respective codons in its protein genes: a proposal for a synonymous codon choice that is optimal for the *E. coli* translational system. J. Mol. Biol. 1981; 151:389–409. 6175758 10.1016/0022-2836(81)90003-6

[35] Elf J. , Nilsson D., Tenson T., Ehrenberg M. Selective charging of tRNA isoacceptors explains patterns of codon usage. Science. 2003; 300:1718–1722. 12805541 10.1126/science.1083811

[36] Lindsley D. , Bonthuis P., Gallant J., Tofoleanu T., Elf J., Ehrenberg M. Ribosome bypassing at serine codons as a test of the model of selective transfer RNA charging. EMBO Rep. 2005; 6:147–150. 15678161 10.1038/sj.embor.7400332 PMC1299242

[37] Dever T.E. , Green R. The elongation, termination, and recycling phases of translation in eukaryotes. Cold Spring Harb. Perspect. Biol. 2012; 4:a013706. 22751155 10.1101/cshperspect.a013706 PMC3385960

[38] Maracci C. , Rodnina M.V. Review: translational GTPases. Biopolymers. 2016; 105:463–475. 26971860 10.1002/bip.22832 PMC5084732

[39] Iwasaki S. , Ingolia N.T. The growing toolbox for protein synthesis studies. Trends Biochem. Sci. 2017; 42:612–624. 28566214 10.1016/j.tibs.2017.05.004 PMC5533619

[40] Avcilar-Kucukgoze I. , Bartholomaus A., Cordero Varela J.A., Kaml R.F., Neubauer P., Budisa N., Ignatova Z. Discharging tRNAs: a tug of war between translation and detoxification in *Escherichia coli*. Nucleic Acids Res. 2016; 44:8324–8334. 27507888 10.1093/nar/gkw697 PMC5041488

