# Supplementary material for: Estimation of peptide elongation times from ribosome profiling spectra
Source: Nucleic Acids Res. 2021 Apr 22;49(9):5124–42. doi: 10.1093/nar/gkab260 (PMC8136808; doi:10.1093/nar/gkab260)
Supplement: gkab260_Supplemental_File [file gkab260_supplemental_file.pdf]

SUPPLEMENTARY INFORMATION

Estimation of peptide elongation times from ribosome profiling spectra

Michael Pavlov, Gustaf Ullman, Zoya Ignatova and Måns Ehrenberg

SUPPLEMENTARY FIGURES

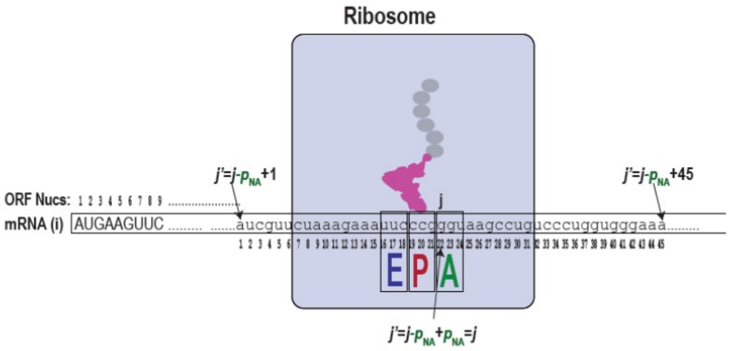

Figure S1. Global and local nucleotide contexts for ribosomal translation of an ORF of type *i*. Global position *j* of the first nucleotide of the A site corresponds to local position $p=p_{NA}=22$ of the first A-site nucleotide. Global nucleotide position *j'* corresponds to local position *p* through $j'=j-p_{NA}+p$, where *p* varies from 1 to $p_{NL}=45$.

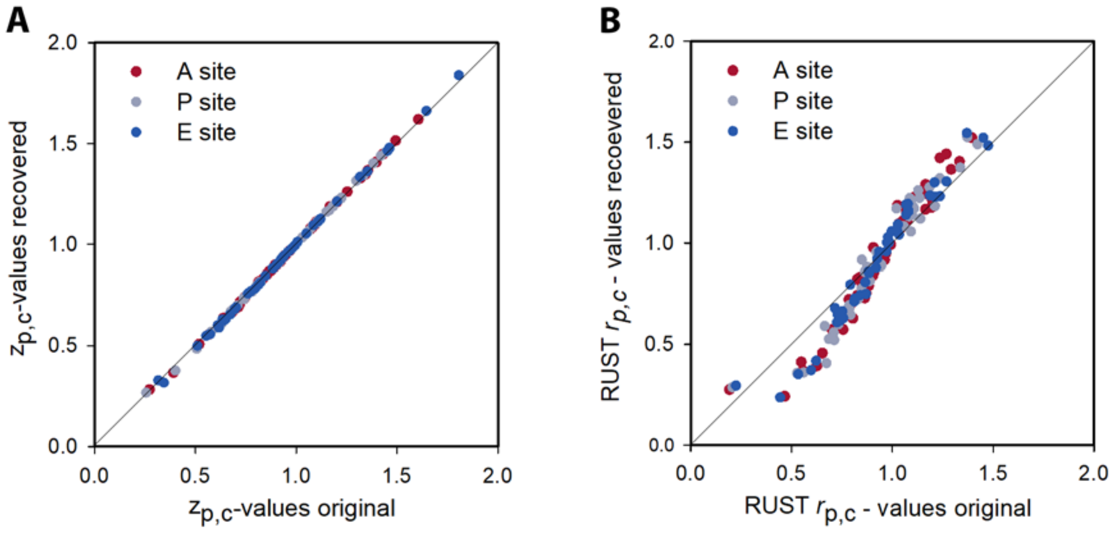

Figure S2. Comparison of original and recovered parameters. (A) Original $z_{p,c}$ values, calculated from an experimental RPF data set containing 945 genes, were used to generate a model dataset; then a new set of $z_{p,c}$ values was recovered from the model dataset; recovered versus original $z_{p,c}$ values for *p*-positions corresponding to the A, P and E site of the local sequence context are plotted. (B) Original RUST ratio metafile table denoted here as $r_{p,c}$ was calculated by implementing the RUST algorithm as described in (1), using the same experimental dataset containing 945 genes as in (A). These original $r_{p,c}$-values were used to generate a model RPF dataset and a new RUST ratio metafile table was recovered from the model data; recovered versus original $r_{p,c}$ parameters for *p*-positions corresponding to the A, P and E site of the local sequence context are plotted.

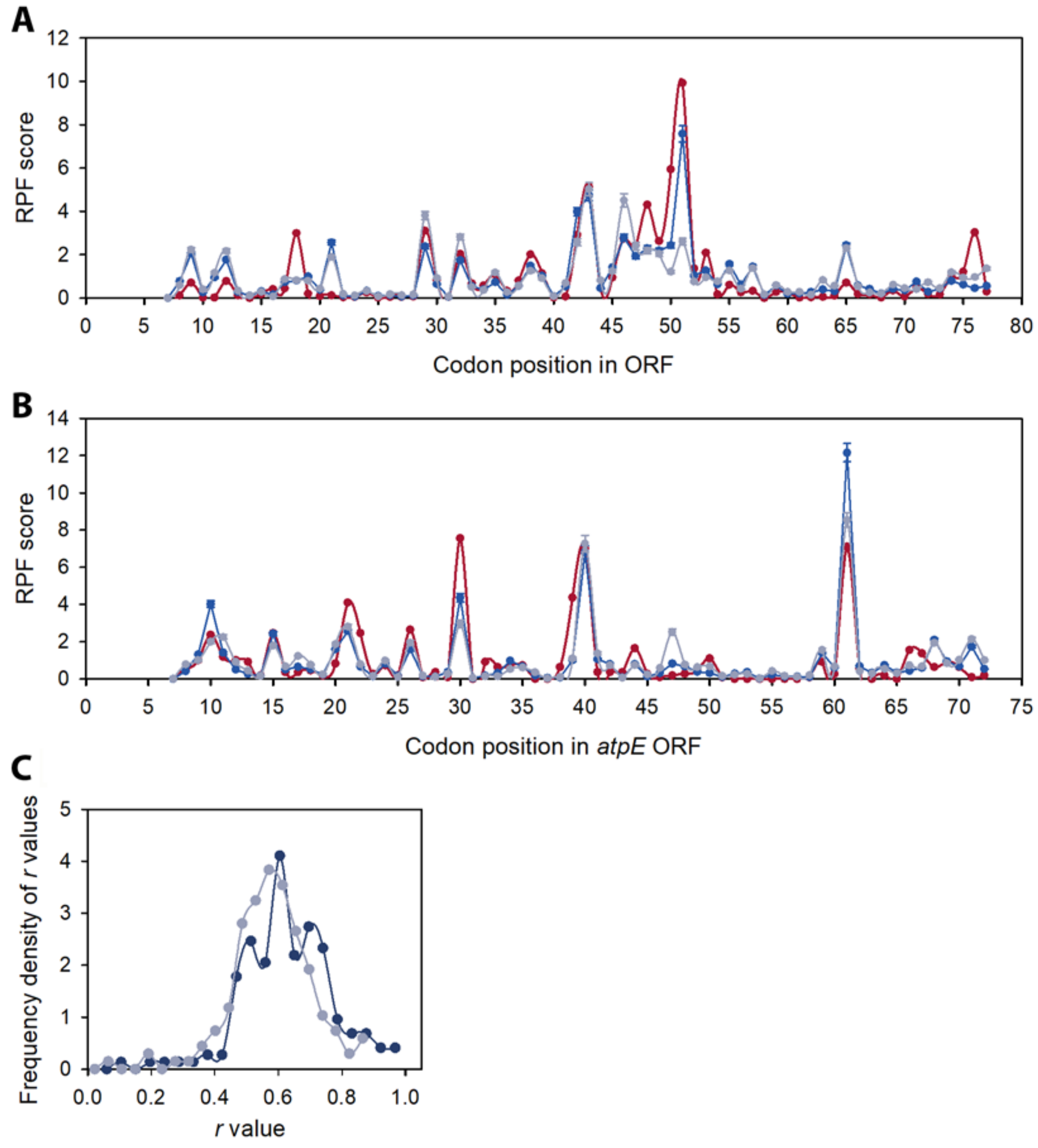

Figure S3. Comparisons of experimental (red), our model derived (blue) and RUST-derived model (light blue) RPF score spectra at codon resolution. (A) Pearson correlation (*r*) between the experimental spectra for *rpsQ* transcript, *r*=0.83 for our model and *r*= 0.63 for RUST. (B) Pearson correlation (*r*) between the experimental spectra for *atpE* transcript *r*=0.81, for our model and *r*=0.8 for RUST. (C) Frequency density of *r* values between experimental spectra and our model spectra (blue) or model RUST spectra (light blue) for 161 highly expressed genes (i.e. with average RPF density $d_i^{\exp} > 5$).

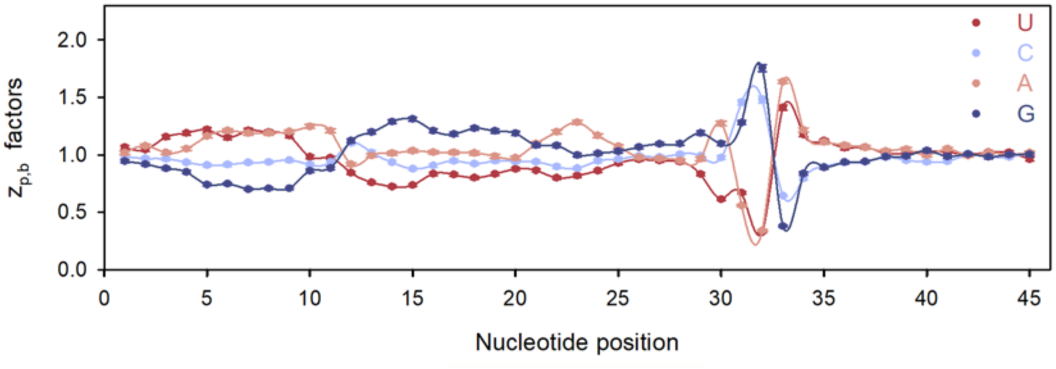

Figure S4. $z_{p.b}$ factors calculated for the standard RPF coverage profile $c_{i,j}^{\exp}$ of *E. coli* AS19 at nucleotide resolution. The $c_{i,j}^{\exp}$ profile was obtained by summation of RPF length-specific coverage profiles $c_{i,j}^{\exp,FL}$ for RPFs with lengths ranging from FL=22 to FL=27 nts.

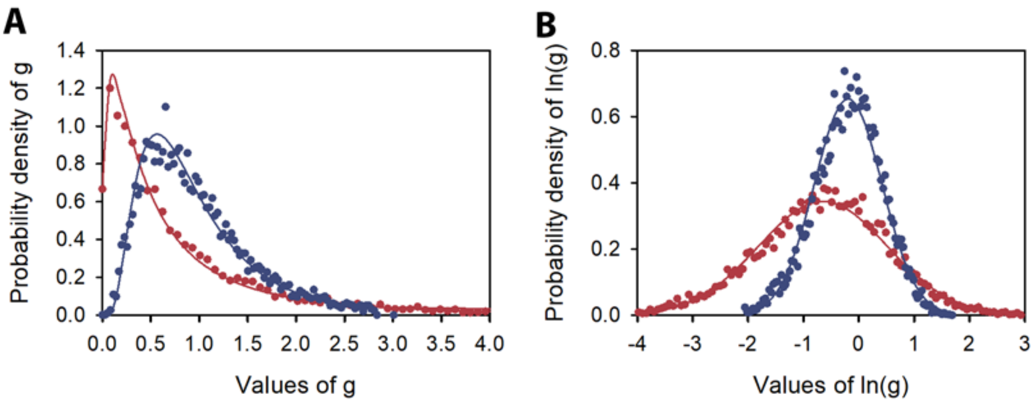

Figure S5. Frequency densities for (A) the values of model g^{mod} (blue) and bias-corrected g^{T} (red) context factors fitted by log-normal probability distribution (solid lines) and (B) the values of the natural logarithms of g^{mod} (blue) and g^{T} (red) context factors fitted by Gaussian probability distribution (solid lines).

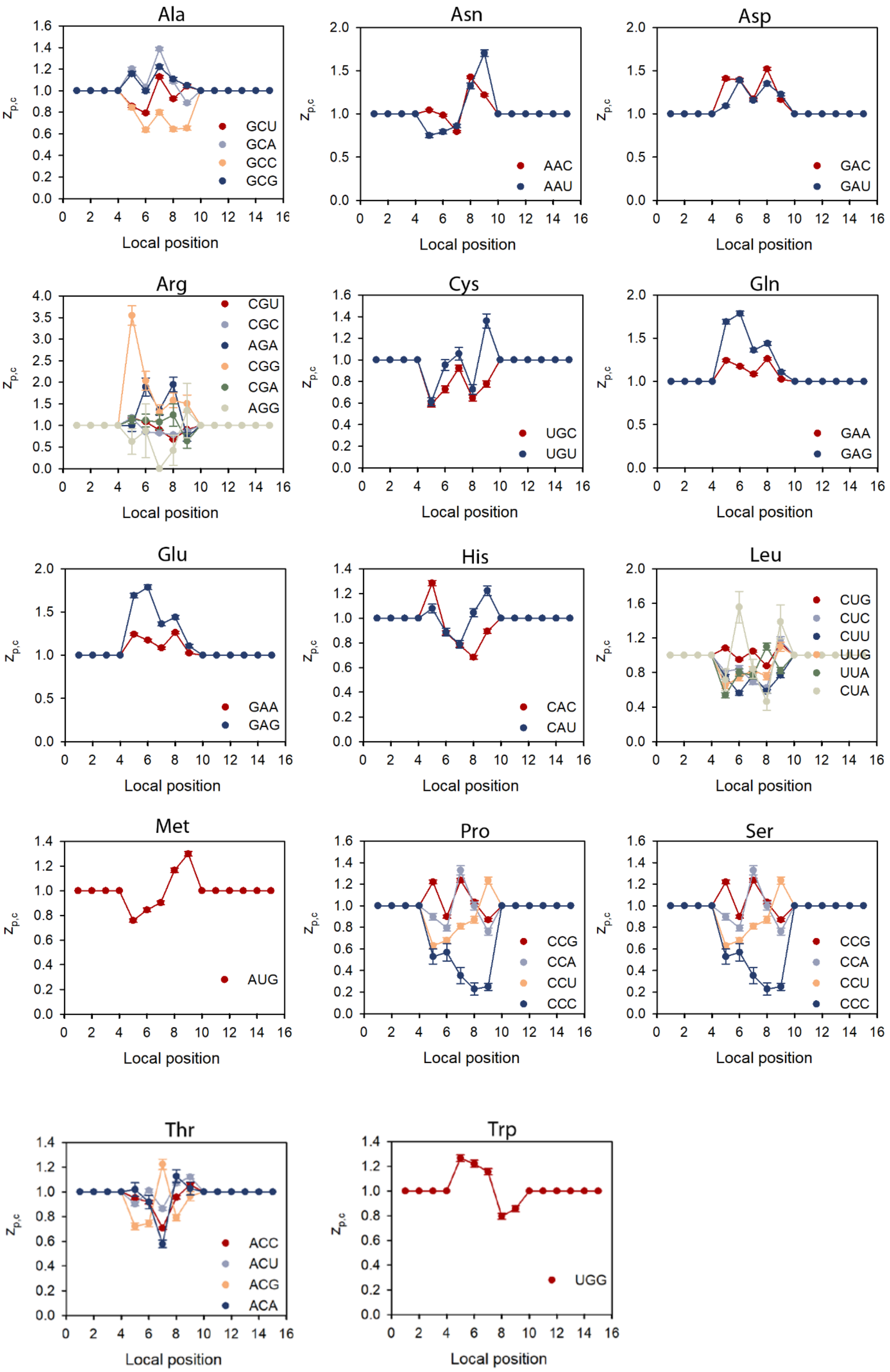
Ala
GCU
GCA
GCC
GCG
Asn
AAC
AAU
Asp
GAC
GAU
Arg
CGU
CGC
AGA
CGG
CGA
AGG
Cys
UGC
UGU
Gln
GAA
GAG
Glu
GAA
GAG
His
CAC
CAU
Leu
CUG
CUC
CUU
UUG
UUA
CUA
Met
AUG
Pro
CCG
CCA
CCU
CCC
Ser
CCG
CCA
CCU
CCC
Thr
ACC
ACU
ACG
ACA
Trp
UGG
$z_{p,c}$
Local position

Figure S6. Variation of $z_{p,c}$ factors in the inner local context positions around the A–site position (p=8) for the codons not included in Figure 7. Large and small $z_{p,c}$-values mean propensity for slow and fast peptide elongation, respectively.

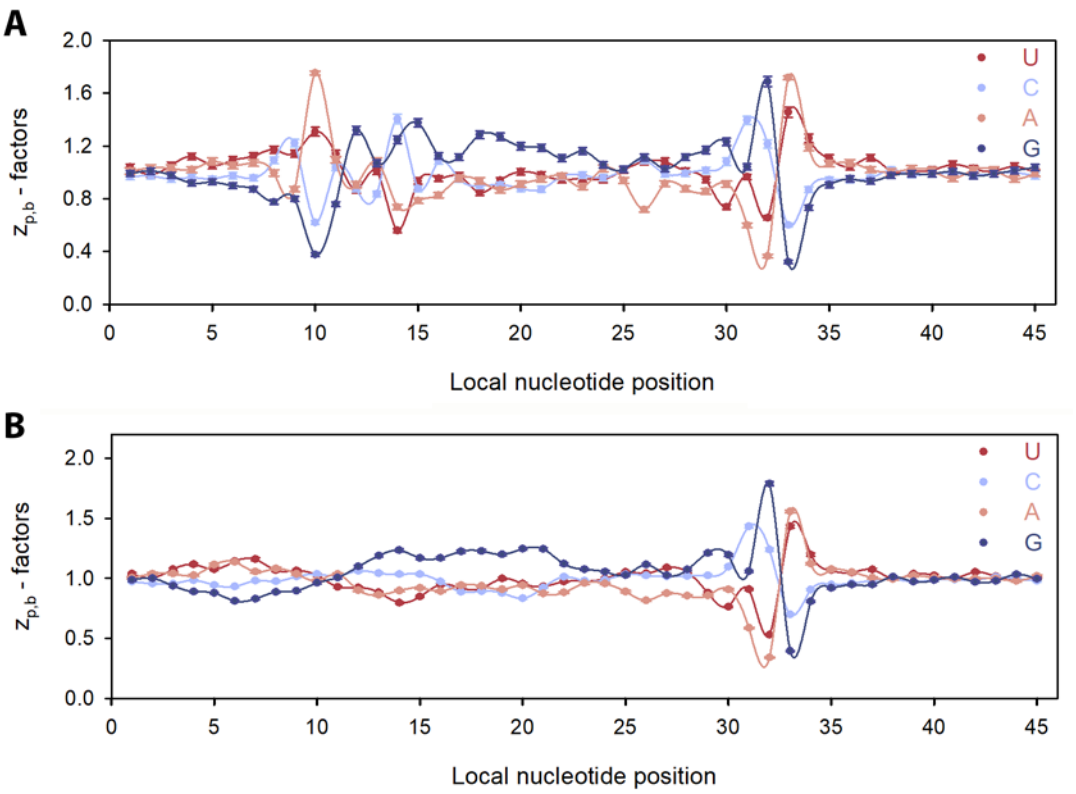

Figure S7. (A) $z_{p.b}^{FL}$ factors for the RPF coverage profile $c_{i,j}^{\exp,FL}$ obtained with RPFs of length FL=23 nts for *E. coli* MG1655, displayed for local nucleotide positions 1 to 45 (see Figure S1); (B) $z_{p,b}$ factors calculated for the standard RPF coverage profile $c_{i,j}^{\exp}$ at nucleotide resolution. The $c_{i,j}^{\exp}$ profile was obtained by summation of RPF length-specific coverage profiles $c_{i,j}^{\exp,FL}$ for RPFs with lengths ranging from FL=22 to FL=27 nts for *E. coli* MG1655; *p*=22 corresponds to the first nucleotide of the local A-site position (Figure S1).

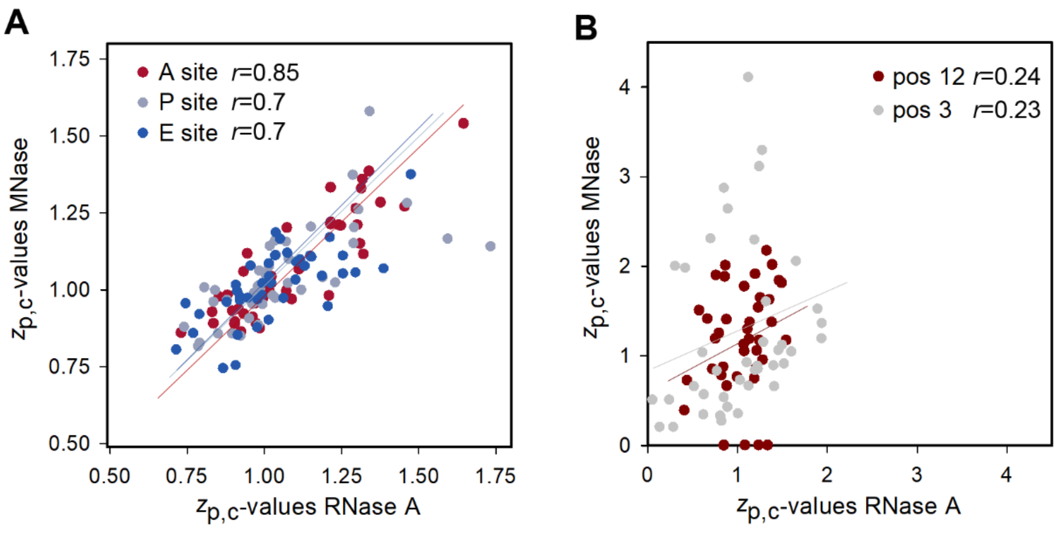

Figure S8. Comparison of $z_{p,c}^{S7}$ and $z_{p,c}^{RA}$ values obtained for the yeast datasets processed with MNase or RNase A for A, P and E positions (A) and for positions 3 and 12 near the edges of the yeast ribosome (B). r, Pearson correlation coefficients calculated for 41 codons with frequency > 0.3%.

SUPPLEMENTARY TEXT

Equations in the main text are always referred to as "Eq. X" and those in the Supplementary Information (SI) as "Eq. XS". For convenience, Eq. 6, which relates factor g_{ij}^{mod} to the local sequence context with current A site at codon position *j* in mRNA of gene *i*, is also provided here in SI format:

$$g_{ij}^{\mathrm{mod}} = \prod_{p=1}^{p_L} z_{p,seq_i(j+p-p_A)} \,. \qquad \text{Eq. 1S}$$

Factors $z_{p,c}$ are the underlying parameters of our model to be determined from the fit of all g_{ij}^{mod}-values to the experimental profiling data. The index $seq_i(j)$ returns the identity, *c*, of a codon at position *j* in ORF$_i$ as an integer in the interval 1 to 64. How model estimated RPF counts c_{ij}^{mod} are related to factors g_{ij}^{mod} and model estimated φ_i -parameters, f_i^{mod}, in Eq. 5 is also reproduced here in SI format:

$$c_{ij}^{\mathrm{mod}} = f_i^{\mathrm{mod}} g_{ij}^{\mathrm{mod}} \,. \qquad \text{Eq. 2S}$$

Maximal log-likelihood function *L*

Using Eq. 2S and notation $C_i^{\exp} = \sum_j c_{ij}^{\exp}$, the log-likelihood function *L* in Eq. 7 can be written as:

$$L = \sum_i \sum_j c_{ij}^{\exp} \ln g_{ij}^{\mathrm{mod}} - \sum_i f_i^{\mathrm{mod}} \sum_j g_{ij}^{\mathrm{mod}} + \sum_i C_i^{\exp} \ln f_i^{\mathrm{mod}} - \sum_i \sum_j \ln(c_{ij}^{\exp}!)) \,. \qquad \text{Eq. 3S}$$

In Eq. 3S and in what follows $\sum_j$ means $\sum_{j=p_A}^{l_i+p_A-p_L}$ implying that *j* in ORF$_i$ runs from codon number p_A to codon number $l_i + p_A - p_L$, where l_i is the total number of codons in ORF$_i$ and p_A is the A-site position in the local codon context of codon length p_L . With $z_{p,c}$ -parameters in Eq. 1S and f_i^{mod} -parameters in Eq. 3S considered as independent variables, the partial derivatives of *L* with respect to f_i^{mod} define the $G_{f_i^{\mathrm{mod}}}$ components of the gradient vector:

$$G_{f_i^{\mathrm{mod}}} = \frac{\partial L}{\partial f_i^{\mathrm{mod}}} = \frac{C_i^{\exp}}{f_i^{\mathrm{mod}}} - \sum_j g_{ij}^{\mathrm{mod}} \,. \qquad \text{Eq. 4S}$$

At maximal *L*, all $G_{f_i^{\mathrm{mod}}}$ components are zero so that:

$$f_i^{\mathrm{mod}} = \frac{C_i^{\exp}}{\sum_j g_{ij}^{\mathrm{mod}}} \,. \qquad \text{Eq. 5S}$$

The partial derivatives of *L* with respect to $z_{p,c}$ define the $G_{z_{p,c}}$ components of the gradient vector:

$$G_{z_{p,c}} = \frac{\partial L}{\partial z_{p,c}} = \sum_i \sum_j c_{ij}^{\exp} \frac{\partial}{\partial z_{p,c}} \ln g_{ij}^{\mathrm{mod}} - \sum_i f_i^{\mathrm{mod}} \sum_j \frac{\partial}{\partial z_{p,c}} g_{ij}^{\mathrm{mod}} \,. \qquad \text{Eq. 6S}$$

It follows from Eq. 1S that:

$$\frac{\partial}{\partial z_{p,c}} g_{ij}^{\mathrm{mod}} = \frac{\partial}{\partial z_{p,c}} \prod_{q=1}^{pL} z_{q,seq_i(q+j-pA)} = \left\{ \prod_{q \neq p}^{pL} z_{q,seq_i(q+j-pA)} \right\} \frac{\partial z_{p,seq_i(p+j-pA)}}{\partial z_{p,c}} \quad . \qquad \text{Eq. 7S}$$

Using that:

$$\frac{\partial z_{p,seq_i(p+j-pA)}}{\partial z_{p,c}} = \delta_{c,seq_i(p+j-pA)} , \qquad \text{Eq. 8S}$$

where $\delta_{c,s}$ is the Kronecker delta introduced in Eq. 8, Eq. 7S simplifies to:

$$\frac{\partial}{\partial z_{p,c}} g_{ij}^{\mathrm{mod}} = \frac{\delta_{c,seq_i(p+j-pA)}}{z_{p,seq_i(p+j-pA)}} \left\{ \prod_{q=1}^{pL} z_{q,seq_i(q+j-pA)} \right\} = \frac{\delta_{c,seq_i(p+j-pA)}}{z_{p,c}} g_{ij}^{\mathrm{mod}} . \qquad \text{Eq. 9S}$$

It also follows that:

$$\frac{\partial}{\partial z_{p,c}} \ln g_{ij}^{\mathrm{mod}} = \frac{1}{g_{ij}^{\mathrm{mod}}} \frac{\partial g_{ij}^{\mathrm{mod}}}{\partial z_{p,c}} = \frac{\delta_{c,seq_i(p+j-pA)}}{z_{p,c}} . \qquad \text{Eq. 10S}$$

Using Eq. 9S and 10S, Eq. 6S can be written as:

$$G_{z_{p,c}} = \frac{1}{z_{p,c}} \left\{ \sum_i \sum_j c_{ij}^{\exp} \delta_{c,seq_i(p+j-pA)} - \sum_i f_i^{\mathrm{mod}} \sum_j g_{ij}^{\mathrm{mod}} \delta_{c,seq_i(p+j-pA)} \right\} . \qquad \text{Eq. 11S}$$

Defining $\Omega_{p,c}^{\exp}$ in Eq. 11S as:

$$\Omega_{p,c}^{\exp} = \sum_i \sum_j C_{ij}^{\exp} \delta_{c,seq_i(j+p-pA)} , \qquad \text{Eq. 12S}$$

and using that maximal *L* requires all the gradient vector components in Eqs 4S and 11S to be zero, one obtains with the help of Eq. 5S:

$$\Omega_{p,c}^{\exp} = \sum_i \frac{C_i^{\exp}}{\sum_j g_{ij}^{\mathrm{mod}}} \sum_j g_{ij}^{\mathrm{mod}} \delta_{c,seq_i(p+j-pA)} . \qquad \text{Eq. 13S}$$

It now follows from Eqs 2S and 5S that model counts c_{ij}^{mod} can be expressed as:

$$c_{ij}^{\mathrm{mod}} = C_i^{\exp} \frac{g_{ij}^{\mathrm{mod}}}{\sum_k g_{ik}^{\mathrm{mod}}} . \qquad \text{Eq. 14S}$$

By analogy with $\Omega_{p,c}^{\exp}$ we define $\Omega_{p,c}^{\mathrm{mod}}$ as:

$$\Omega_{p,c}^{\mathrm{mod}} = \sum_i \sum_j c_{ij}^{\mathrm{mod}} \delta_{c,seq_i(p+j-pA)} . \qquad \text{Eq. 15S}$$

Eq. 13S can now be re-written as:

$$\Omega_{p,c}^{\exp} = \sum_i \sum_j C_{ij}^{\mathrm{mod}} \delta_{c,seq_i(p+j-pA)} \equiv \Omega_{p,c}^{\mathrm{mod}} . \qquad \text{Eq. 16S}$$

Eq. 16S implies that *L* is maximized for a set of $z_{p,c}$ parameters in which model (Eq. 15S) and experimental (Eq. 16S) fingerprints coincide. We note that from Eq. 14S follows that:

$$C_i^{\mathrm{mod}} = \sum_j c_{ij}^{\mathrm{mod}} = C_i^{\exp} \qquad \text{Eq. 17S}$$

This means that the fulfillment of maximum likelihood conditions imposed by Eq. 5S implies that the total per gene RPF counts are conserved, i.e. that $C_i^{\text{mod}} = C_i^{\text{exp}}$.

We further note that Eq. 13S, valid for a set of g_{ij}^{mod} and a set of c_{ij}^{exp} values, remains valid for the same set of g_{ij}^{mod} values in conjunction with any set $\alpha \cdot c_{ij}^{\text{exp}}$ of re-scaled c_{ij}^{exp} values. This is because such a scaling affects equally $\Omega_{p,c}^{\text{exp}}$ on the left and C_i^{exp} on the right side of Eq. 13S.

Reduced log-likelihood function L^r

Since Eq. 5S is valid for maximal *L*, f_i^{mod} in Eq. 3S can be substituted by $C_i^{\text{exp}} / \sum_j g_{ij}^{\text{mod}}$, to obtain a "reduced" log likelihood function L^r depending only on g_{ij}^{mod}-values, and, hence, through Eq. 1S, being completely defined by the $z_{p,c}$-factors:

$$L^r = \sum_i \sum_j c_{ij}^{\text{exp}} \ln g_{ij}^{\text{mod}} - \sum_i \frac{C_i^{\text{exp}}}{\sum_j g_{ij}^{\text{mod}}} \sum_j g_{ij}^{\text{mod}} + \sum_i C_i^{\text{exp}} \ln \frac{C_i^{\text{exp}}}{\sum_j g_{ij}^{\text{mod}}} - \sum_{ij} \ln(c_{ij}^{\text{exp}}!)) =$$
$$= \sum_i \sum_j c_{ij}^{\text{exp}} \ln g_{ij}^{\text{mod}} - \sum_i C_i^{\text{exp}} + \sum_i C_i^{\text{exp}} \ln C_i^{\text{exp}} - \sum_i C_i^{\text{exp}} \ln\left(\sum_j g_{ij}^{\text{mod}}\right) - \sum_{ij} \ln(c_{ij}^{\text{exp}}!)). \quad \text{Eq. 18S}$$

For the components, $G_{z_{p,c}}^r$, of the gradient of L^r one obtains:

$$G_{z_{p,c}}^r = \frac{\partial L^r}{\partial z_{p,c}} = \sum_{ij} c_{ij}^{\text{exp}} \frac{\partial}{\partial z_{p,c}} \ln g_{ij}^{\text{mod}} - \sum_i C_i^{\text{exp}} \frac{\partial}{\partial z_{p,c}} \ln\left(\sum_j g_{ij}^{\text{mod}}\right) =$$
$$= \sum_{ij} c_{ij}^{\text{exp}} \frac{\partial}{\partial z_{p,c}} \ln g_{ij}^{\text{mod}} - \sum_i \frac{C_i^{\text{exp}}}{\sum_j g_{ij}^{\text{mod}}} \sum_j \frac{\partial}{\partial z_{p,c}} g_{ij}^{\text{mod}}. \quad \text{Eq. 19S}$$

From Eqs. 9S, 10S and 13S it also follows that:

$$G_{z_{p,c}}^r = \frac{1}{z_{p,c}} \left\{ \Omega_{p,c}^{\text{exp}} - \sum_i \frac{C_i^{\text{exp}}}{\sum_j g_{ij}^{\text{mod}}} \sum_j g_{ij}^{\text{mod}} \delta_{c,seq_i(p+j-pA)} \right\}. \quad \text{Eq. 20S}$$

It is seen that the condition of zero gradient components in Eq. 20S leads exactly to Eq. 13S, the latter assuring zero gradient components also of *L*. This proves that Eq. 13S is fulfilled at the point in $z_{p,c}$-parameter space where the gradient of L^r is zero and L^r maximized.

To actually find the set of $z_{p,c}$-parameters that maximize L^r, we use a Levenberg-Marquardt type algorithm (2,3) that uses the Hessian of L^r. It first finds the global maximum of L^r in a series of steps by solving the equation:

$$\vec{G}^r(\vec{z}_k) = \left\{ \hat{H}^r(\vec{z}_k) + \lambda_k \cdot diag(\hat{H}^r(\vec{z}_k)) \right\} \vec{d}_k \quad \text{Eq. 21S}$$

for the directional vector $\vec{d}_k$ and then stepping to $\vec{z}_{k+1}$ from $\vec{z}_k$ in $z_{p,c}$-parameter space through $\vec{z}_{k+1} = s_k \vec{d}_k + \vec{z}_k$. Here, $\hat{H}^r(\vec{z}_k)$ and $\vec{G}^r(\vec{z}_k)$ are the current Hessian and the gradient of L^r,

respectively; λ_k is the Levenberg-Marquardt parameter, which in every iteration is chosen as described (3) and the length, s_k , in step number *k* is determined from the condition $(\vec{G}^r(\vec{z}_k + s_k\vec{d}_k), \vec{d}_k) = 0$, i.e. that the direction of the gradient at $\vec{z}_{k+1}$ is perpendicular to $\vec{d}_k$.

Hessian matrix of the reduced log-likelihood function

For the Hessian of L^r one easily gets:

$$H^r_{p,c;\tilde{p},\tilde{c}} = \frac{\partial}{\partial z_{\tilde{p},\tilde{c}}} G^r_{z_{p,c}} = \frac{\partial}{\partial z_{\tilde{p},\tilde{c}}} \frac{1}{z_{p,c}} \left\{ \Omega^{\exp}_{p,c} - \sum_i \frac{C_i^{\exp}}{\sum_j g_{ij}^{\mathrm{mod}}} \sum_j g_{ij}^{\mathrm{mod}} \delta_{c,seq_i(p+j-pA)} \right\} =$$

$$= -\frac{\delta_{c,\tilde{c}}\delta_{p,\tilde{p}}}{z^2_{p,c}} \frac{1}{z_{p,c}} \left\{ \Omega^{\exp}_{p,c} - \sum_i \frac{C_i^{\exp}}{\sum_j g_{ij}^{\mathrm{mod}}} \sum_j g_{ij}^{\mathrm{mod}} \delta_{c,seq_i(p+j-pA)} \right\} - \qquad \text{Eq. 22S}$$

$$-\frac{1}{z_{p,c}} \left\{ \frac{\partial}{\partial z_{\tilde{p},\tilde{c}}} \sum_i \frac{C_i^{\exp}}{\sum_j g_{ij}^{\mathrm{mod}}} \sum_j g_{ij}^{\mathrm{mod}} \delta_{c,seq_i(p+j-pA)} \right\}.$$

Eq. 9S in conjunction with algebraic reshuffling leads to:

$$H_{p,c;\tilde{p},\tilde{c}} = -\frac{\delta_{c,\tilde{c}}\delta_{p,\tilde{p}}}{z_{p,c}} G_{z_{p,c}} + \frac{1}{z_{p,c} z_{\tilde{p},\tilde{c}}} \left\{ \sum_i U_i^{\exp} \frac{\Theta^{\mathrm{mod}}_{p,c,i}}{G_i^{\mathrm{mod}}} \frac{\Theta^{\mathrm{mod}}_{\tilde{p},\tilde{c},i}}{G_i^{\mathrm{mod}}} \right\} -$$

$$-\frac{1}{z_{p,c} z_{\tilde{p},\tilde{c}}} \left\{ \sum_i \frac{C_i^{\exp}}{G_i^{\mathrm{mod}}} \sum_j \delta_{c,seq_i(p+j-pA)} \delta_{\tilde{c},seq_i(\tilde{p}+j-pA)} g_{ij}^{\mathrm{mod}} \right\}. \qquad \text{Eq. 23S}$$

Here, to simplify we have introduced notations:

$$G_i^{\mathrm{mod}} = \sum_j g_{ij}^{\mathrm{mod}} \qquad \text{Eq. 24S}$$

and

$$\Theta^{\mathrm{mod}}_{p,c,i} = \sum_j \delta_{c,seq_i(p+j-pA)} g_{ij}^{\mathrm{mod}} . \qquad \text{Eq. 25S}$$

We note that for any position *p* of the local context the corresponding *p*-row of the $z_{p,c}$ -factors table can be multiplied by any number α_p with the validity of Eq. 13S retained. To remove this scaling ambiguity we set $z_{p,1} = 1$ for all positions *p*, meaning that the search for maximal L^r explores only a subspace of dimension $p_L \times (N_c - 1)$ (where N_c=61 is the number of sense codon types) of the total $z_{p,c}$ -parameter space of $p_L \times N_c$ dimension. In this subspace the Hessian matrix can be inverted, which leads to a unique solution for the global L^r -maximum.

We also note that $a \cdot c_{ij}^{\exp}$ scaling affects the errors of the $z_{p,c}$ -parameter estimates (but not their expected values). The reason is that by such scaling the components of both gradient and Hessian will be multiplied by a factor *a*, meaning that the parameter errors computed from the main diagonal of the

inverted Hessian, $H^{-1}_{p,c;p,c}$, will be multiplied by a factor $\sqrt{a}$. Accordingly, when the scaling factor *a* for the RPF counts is unknown, only the relative errors of the $z_{p,c}$ parameters can be obtained from $H^{-1}_{p,c;p,c}$. To speed up computations, we use an approximate Hessian $H^{appr}_{p,c;\tilde{p},\tilde{c}}$ obtained by discarding the cross-position terms (i.e. terms with $\tilde{p} \neq p$) in $H_{p,c;\tilde{p},\tilde{c}}$ given by Eq.23. $H^{appr}_{p,c;\tilde{p},\tilde{c}}$ is given by:

$$H^{appr}_{p,c;\tilde{p},\tilde{c}} = -\frac{\delta_{p,\tilde{p}}\delta_{c,\tilde{c}}}{z^2_{p,c}}\Omega^{\exp}_{p,c} + \frac{\delta_{p,\tilde{p}}}{z_{p,c}z_{p,\tilde{c}}}\left\{\sum_i C^{\exp}_i \frac{\Theta^{\mathrm{mod}}_{p,c,i}}{G^{\mathrm{mod}}_i}\frac{\Theta^{\mathrm{mod}}_{p,\tilde{c},i}}{G^{\mathrm{mod}}_i}\right\}, \qquad \text{Eq. 26S}$$

so that $H^{appr}_{p,c;p,\tilde{c}} = H_{p,c;p,\tilde{c}}$. The increase in computation speed comes from the omission of a time consuming calculation of the last term in Eq. 23S and from the fact that the matrix form of $H^{appr}_{p,c;\tilde{p},\tilde{c}}$ is now a block-diagonal matrix with p_L blocks of $(N_c - 1)\times(N_c - 1)$ size. The latter property allows for a fast and efficient solution of Eq. 21S. The speed of finding zero gradient components by solving Eq. 13 increases, despite the increase in total number of algorithm iteration steps in comparison with when the complete Hessian of Eq. 23S is used.

One position model and initial guesses for the $z_{p,c}$ parameter table

Let us consider a model for which $z_{p,c}$ factors in Eq. 1S vary with *c* for the position *p* only, while the rest of $z_{q,c}$ factors with $q \neq p$ are set to 1. Eq. 1S reduces then to:

$$g^{\mathrm{mod}}_{ij} = \hat{z}_{p,seq_i(j+p-p_A)} \qquad \text{Eq. 27S}$$

We put the "hat" symbol over z-parameters in Eq. 27S to indicate explicitly that the set of $\hat{z}_{p,c}$ refers to the special case of the general model in which only one position *p* matters. It follows then that here Eq. S13, which defines the maximum of the likelihood function, can be re-cast as:

$$\hat{z}_{p,c} = \frac{\Omega^{\exp}_{p,c}}{\sum_i \frac{C^{\exp}_i}{\sum_c \hat{z}_{p,c} n_{i,p,c}} n_{i,p,c}}, \qquad \text{Eq. 28S}$$

where

$$n_{i,p,c} = \sum_{j=p_A}^{l_i - p_L + p_A} \delta_{c,seq_i(p+j-p_A)} = \sum_{k=p}^{l_i - p_L + p} \delta_{c,seq_i(k)} \qquad \text{Eq. 29S}$$

It is seen that $n_{i,p,c}$ is the number of codons of type c in the part of ORF$_i$ that starts from codon number *p* and ends at codon number $l_i - p_L + p$. It follows then that $n_{i,p,c}$ can vary slightly with *p* for codons close to the boundaries of ORF$_i$. Eq. 28S is easily solved by the following iterations starting from $\hat{z}_{p,c} = 1$:

$$\hat{z}_{p,c}^{next} = \frac{\Omega_{p,c}^{\exp}}{\sum_i \frac{C_i^{\exp}}{\sum_c \hat{z}_{p,c}^{previous} n_{i,p,c}} n_{i,p,c}} \qquad \text{Eq. 30S}$$

We note that, like in the general case, if some $\hat{z}_{p,c}$ set is a solution of Eq. 28S then for any scaling factor $\alpha \cdot \hat{z}_{p,c}$ is also a solution. Here, this "scaling uncertainty" is removed by rescaling $\hat{z}_{p,c}^{next}$ after each iteration to satisfy the equality:

$$\sum_i \frac{U_i^{\exp}}{\sum_c \hat{z}_{p,c}^{next} n_{i,p,c}} = \sum_i \frac{U_i^{\exp}}{n_i}, \qquad \text{Eq. 31S}$$

Here, $n_i = l_i - p_L + 1$ is the number of codons in the "inner" region of ORF$_i$. The solution stabilizes to $\hat{z}_{p,c}^{next} = \hat{z}_{p,c}^{previous}$ within the tolerance of 10^{-10} after just a few iterations.

It is clear that by solving Eq. 28S for each position from p=1 to p=p_L one obtains a $\hat{z}_{p,c}$ parameter table of the same dimensions ($p_L \times N_c$) as for the general model in Eq. 1S. We use the $\hat{z}_{p,c}$ parameter table as the initial guess for $z_{p,c}$ parameters in our ML algorithm. We note that the $\hat{z}_{p,c}$ parameter set differs from the $z_{p,c}$ parameter set obtained as the solution of Eq. 13S for general model in Eq. 1S and that the $\hat{z}_{p,c}$ parameters do not maximize the likelihood function for the general model. Still $\hat{z}_{p,c}$ table is useful if one wishes to roughly estimate the S_p position sensitivity (e.g. by using $\hat{z}_{p,c}$ instead of $z_{p,c}$ in Eq. 18).

Self-consistency of the maximum-likely hood approach

The extremum condition of Eq. 13S ensures self-consistency of the present modeling approach in the following sense. Suppose that a set of $z_{p,c}$ parameters that generate the set of g_{ij}^{mod} parameters satisfying Eq. 13S has been found. Suppose further that this set of g_{ij}^{mod} parameters is used to obtain model RPF counts c_{ij}^{mod} according to Eq. 14S and that this model c_{ij}^{mod} set is used to replace the experimental RPF dataset $c_{ij}^{\exp}$ in Eq. 12S. It follows then that the original Eq. 13S is reproduced because Eqs 16S and 17S ensure both that $\Omega_{p,c}^{\exp} = \Omega_{p,c}^{\mathrm{mod}}$ and that $C_i^{\exp} = C_i^{\mathrm{mod}}$. Hence, the same set of originally obtained g_{ij}^{mod} parameters and underlying $z_{p,c}$ parameters will be recovered when Eq.13 is solved for the model data set.

Standard scaling of $z_{p,c}$ parameter sets

We note that although the condition $z_{p,1} = 1$ is adequate for implementation of our ML-algorithm, it is inappropriate for comparing $z_{p,c}$ factors for the same codon *c* at different positions *p* in the local context. To make such a comparison meaningful we re-scale each row *p* of the $z_{p,c}$ parameter table obtained by the algorithm in such a way that in the final table the weighted average $\overline{z}_p$ of $z_{p,c}$ along each table row is one, i.e.:

$$\overline{z}_p = \sum_c w_{p,c} z_{p,c} = 1. \qquad \text{Eq. 32S}$$

Here, the $w_{p,c}$ weights are chosen as:

$$w_{p,c} = \frac{\sum_i f_i^{\mathrm{mod}} n_{i,p,c}}{\sum_i f_i^{\mathrm{mod}} n_i}. \qquad \text{Eq. 33S}$$

We also note that for $n_{i,p,c}$ (see Eq. 29S):

$$\sum_c n_{i,p,c} = \sum_{k=p}^{l_i - p_L + p} \sum_c \delta_{c,seq_i(k)} = l_i - p_L + 1 = n_i . \qquad \text{Eq. 34S}$$

It then follows from Eqs 32S and 34S that $\sum_c w_{p,c} = 1$, a condition providing the proper normalization of Eq. 32S. We note that $n_{i,p,c}$ defined by Eq. 29S varies but slightly with *p* for most ORFs. Furthermore, since f_i^{mod} is proportional to the expression level of gene *i* the products $f_i^{\mathrm{mod}} n_{i,p,c}$ in Eq. 33S are proportional to the frequency with which the ribosome encounters codons of type *c* in the inner region of the ORF. The rationale behind the definition of weights, $w_{p,c}$ by Eq. 33S is that they confer higher statistical weight to codons that are frequent both in the genome and on the codon translating ribosome.

Probability distributions of RPFs

Probability distribution of RNA fragment copy numbers. The probability p_{ij} that a particular ribosome is at codon (*i,j*) by abrupt sample freezing and will generate a footprint for further PCR amplification is given by:

$$p_{ij} = \frac{v_i \tau_{ij} \beta_{ij}}{\sum_{k,l} v_k \tau_{kl}} \qquad \text{Eq. 35S}$$

The denominator summation in Eq. 35S is over all transcriptome positions (*k,l*); v_i is the frequency of translation initiation of transcripts of type *i*, τ_{ij} the expected dwell time on codon *j* of a transcript

(gene) of type *i* and β_{ij} the probability that the segment protected by the ribosome with the A site at position (*i,j*) is successfully processed to a fragment ready for PCR-amplification (i.e. cleaved out by a nuclease and ligated). When there are M translating ribosomes the average number λ_{ij} of generated (*i,j*) fragments is given by

$$\lambda_{ij} = Mp_{ij} \qquad \text{Eq. 36S}$$

Depending on the strength of gene expression (ν_i), length of dwell time (τ_{ij}) and probability (β_{ij}) of nuclease cleavage and ligation of a ribosome covered mRNA segment, the average values (λ_{ij}) vary from numbers near 0 to many thousands. In repeated, identical experiments with M translating ribosomes in the cell population, the copy numbers n_{ij} of fragments with (*i,j*) coordinate display a relative variation around its expected value λ_{ij} which increases with decreasing λ_{ij}. Each such experiment can be considered as a series of "M" Bernoulli trials, where the outcome for each is to generate an *(i,j)* fragment or not. It then follows that the number n_{ij} of generated (*i,j*) fragments is binomially distributed:

$$Bin(n_{ij} \mid M, p_{ij}) = \frac{M!}{\left(M - n_{ij}\right)! n_{ij}!} \left(p_{ij}\right)^{n_{ij}} \left(1 - p_{ij}\right)^{M - n_{ij}} \qquad \text{Eq. 37S}$$

Since, for all (*i,j*), p_{ij}<<1 and n_{ij} <<M, the Binomial distribution in Eq. 37S is for each copy number n_{ij} approximated by a Poisson distribution:

$$Po(n_{ij} \mid \lambda_{ij}) = \frac{\left(\lambda_{ij}\right)^{n_{ij}}}{n_{ij}!} e^{-\lambda_{ij}} \qquad \text{Eq. 38S}$$

In sum, the copy number n_{ij} of (*i,j*) fragments before PCR amplification is Poisson distributed with both expected value and variance equal to λ_{ij}.

<u>Probability distribution of DNA fragment copy numbers after PCR amplification</u>. After amplification each copy number n_{ij} is increased by a factor A_{ij}. The probability that there are $n_{ij}^A = A_{ij} n_{ij}$ fragments of type *(i,j)* is given by a rescaled version of the Poisson distribution in Eq.38S:

$$Po(n_{ij}^A \mid \lambda_{ij}) = \frac{\lambda_{ij}^{(n_{ij}^A / A_{ij})}}{(n_{ij}^A / A_{ij})!} e^{-\lambda_{ij}} \quad , \qquad \text{Eq. 39S}$$

or equivalently:

$$Po(A_{ij} n_{ij} \mid \lambda_{ij}) = \frac{\left(\lambda_{ij}\right)^{n_{ij}}}{n_{ij}!} e^{-\lambda_{ij}} \qquad \text{Eq. 40S}$$

Probability distribution of DNA fragment copy numbers in aliquots for Illumina sequencing. In our experiments a small fraction, q, of a PCR amplified library (normally 1-0.1%) is sequenced. Thus, the probability for a number m_{ij} of fragments in the sequenced library aliquot, conditional on the presence of $A_{ij} n_{ij}$ fragments in the whole amplified library, is also of Poisson type with expected value $\theta_{ij} = qA_{ij} n_{ij}$:

$$Po(m_{ij} \mid \theta_{ij} = qA_{ij} n_{ij}) = \frac{\left(qA_{ij} n_{ij}\right)^{m_{ij}} e^{-qA_{ij} n_{ij}}}{m_{ij}!} \qquad \text{Eq. 41S}$$

The unconditional probability that there are m_{ij} fragments on the chip of the sequencing instrument is then given by multiplying the conditional probability in Eq. 41S with the probability $Po(A_{ij} n_{ij} \mid \lambda_{ij})$ of $A_{ij} n_{ij}$ fragments in Eq. 40S and summing over all n_{ij}-values:

$$P(m_{ij} \mid \lambda_{ij}, qA_{ij}) = \sum_{n_{ij}=0}^{\infty} \frac{\left(qA_{ij} n_{ij}\right)^{m_{ij}} e^{-qA_{ij} n_{ij}}}{m_{ij}!} \frac{\left(\lambda_{ij}\right)^{n_{ij}}}{n_{ij}!} e^{-\lambda_{ij}} \qquad \text{Eq. 42S}$$

The probability distribution in Eq. 8 is of Neyman type A (NA):

$$P_{NA}(m_{ij} \mid \lambda_{ij}, v_{ij} = qA_{ij}) = \frac{v_{ij}^{m_{ij}} e^{-\lambda_{ij}}}{m_{ij}!} \sum_{n=0}^{\infty} \frac{n^{m_{ij}} \lambda_{ij}^{n} e^{-v_{ij} n}}{n!} \qquad \text{Eq. 43S}$$

with parameters λ_{ij}, $v_{ij} = qA_{ij}$. The expected value λ_{Sij} and variance σ_{Sij}^2 for m_{ij} distributed according to Eq. 43S are:

$$\lambda_{Sij} = \lambda_{ij} \cdot qA_{ij}, \; \sigma_{Sij}^2 = \lambda_{ij} \cdot qA_{ij}(1 + qA_{ij}) \qquad \text{Eq. 44S}$$

It is seen that in the case of uniform PCR amplification (i.e. when $A_{ij} = A$ for all (*i,j*)), the original expected average and variance values, both equal to λ_{ij}, are uniformly scaled by factors qA and $qA(1+qA)$, respectively. This means that the m_{ij}-counts are over-dispersed compared to Poisson and that when $qA < 1$ the distribution in Eq. 43S is close to its Poisson limit with $\lambda_{Sij} = \lambda_{ij} \cdot qA$ (4). From this analysis follows that the sampling procedure for the sequencing of a small fraction of reads from the PCR-amplified libraries brings the distribution of the reads much closer to a Poisson distribution than the distribution just after the amplification step. The condition $qA < 1$ was satisfied in our profiling experiments supporting the relevance our statistical ML-approach.

Finally, the Neyman type A distribution can be emulated by a Negative Binomial (Polya) distribution with the same expected value λ_{Sij} and variance σ_{Sij}^2 as in Eq. 44S:

$$P_{NB}(m_{ij} \mid \lambda_{ij}, p = \frac{1}{1+qA_{ij}}) = \frac{\Gamma(\lambda_{ij} + m_{ij})}{\Gamma(\lambda_{ij}) m_{ij}!} p^{\lambda_{ij}} (1-p)^{m_{ij}}, \qquad \text{Eq. 45S}$$

where $\Gamma(x)$ is the standard Gamma function that extends factorial to real numbers. Using that $\Gamma(x) = (x-1)\Gamma(x-1)$ one can eliminate $\Gamma(x)$ in Eq.45S and rewrite it in a simpler form:

$$P_{NB}(m_{ij}) = \frac{\lambda_{ij}}{m_{ij} + \lambda_{ij}} \left(\frac{1}{1+qA_{ij}} \right)^{\lambda_{ij}} \left(1 - \frac{1}{1+qA_{ij}} \right)^{m_{ij}} \cdot \prod_{k=1}^{m_{ij}} (1 + \frac{\lambda_{ij}}{k}) \qquad \text{Eq.46S}$$

Equivalence of protein-number and protein-mass descriptions under conditions of exponential (balanced) cell growth

Let us consider the dynamics of protein number fractions $y_i = P_i / \sum_k P_k$:

$$\frac{d}{dt} y_i = \frac{1}{\sum_k P_k} \frac{d}{dt} P_i - y_i \frac{1}{\sum_k P_k} \sum_k \frac{d}{dt} P_k = \frac{1}{P_{tot}} \frac{d}{dt} P_i - \mu_P \cdot y_i \qquad \text{Eq. 47S}$$

Here we introduced $P_{tot} = \sum_k P_k$ and took into account that:

$$\mu_P = \frac{1}{P_{tot}} \frac{dP_{tot}}{dt} = \frac{1}{\sum_k P_k} \sum_k \frac{d}{dt} P_k = \frac{\gamma P_R}{P_{tot}} \sum_i \frac{u_i}{T_i^{\text{mod}}} \qquad \text{Eq. 48S}$$

That the growth rate μ_P is defined through protein copy number and the last equality in Eq. 48S follows from Eq. 33 of the main text. Further, taking into account Eq. 35 in the main text, Eq. 47S can be written as:

$$\frac{d}{dt} y_i = \frac{1}{P_{tot}} \frac{\gamma P_R}{C_{tot}^T t_e} f_i^{\text{mod}} - y_i \frac{\gamma}{t_e} \frac{P_R}{P_{tot}} \frac{1}{C_{tot}^T} \sum_k f_k^{\text{mod}} \qquad \text{Eq. 49S}$$

During exponential growth the copy number fraction, y_i, of protein *i* in the proteome remains constant so that $dy_i / dt = 0$. It then follows from Eq.49S that:

$$y_i = \frac{P_i}{P_{tot}} = \frac{f_i^{\text{mod}}}{\sum_k f_k^{\text{mod}}} \qquad \text{Eq. 50S}$$

This means, in particular, that $P_R / P_{tot} = f_R^{\text{mod}} / \sum_i f_i^{\text{mod}}$ and, in general, that $P_i / P_j = f_i^{\text{mod}} / f_j^{\text{mod}}$, which justifies Eq. 37 in the main text.

To show that the growth rate equation based on total protein copy number (Eq. 48S) is equivalent to that based on total protein mass under condition of exponential growth, we introduce the length L_i of protein *i* in amino acids and re-write Eq. 33 from the main text for rate of the protein numbers P_i increase as :

$$\frac{d}{dt} L_i P_i = \gamma P_R \frac{u_i}{T_i^{\text{mod}} / L_i} \qquad \text{Eq. 51S}$$

Here, P_R is the current number ribosomes in the population, γ the ribosome fraction in elongation phase, estimated as 0.8 by Dennis and Bremer (5) and u_i is the fraction of elongating ribosomes devoted to synthesis of proteins i.

Let us introduce the relative mass, $M_i = L_i P_i$ as the total number of peptide bonds in all proteins *i* in the cell population, and the average time of peptide bond formation in protein *i* as, $\tau_i = T_i^{\text{mod}} / L_i$. We can then re-write Eq. 51S as:

$$\frac{d}{dt} M_i = \gamma P_R \frac{u_i}{\tau_i} \qquad \text{Eq. 52S}$$

The growth rate defined as relative increase in the total number of peptide bonds, $M = \sum_i M_i$, in proteome (6) can then be expressed as:

$$\mu_M = \frac{1}{M} \sum_i M_i = \gamma \frac{P_R}{M} \sum_i \frac{u_i}{\tau_i} \qquad \text{Eq. 53S}$$

To prove that the growth rate μ_M in Eq. 53S is the same as μ_P in Eq. 48S we need to show that:

$$\mu_P = \frac{\gamma P_R}{P_{tot}} \sum_i \frac{u_i}{T_i^{\text{mod}}} = \frac{\gamma P_R}{M} \sum_i \frac{u_i}{\tau_i} = \mu_M \qquad \text{Eq. 54S}$$

To this end we note now that using Eq. 33 of the main text Eq. 49S can be rewritten as:

$$\frac{d}{dt} y_i = \gamma y_R \frac{u_i}{T_i^{\text{mod}}} - y_i \gamma y_R \sum_k \frac{u_k}{T_k^{\text{mod}}} \qquad \text{Eq. 55S}$$

Since during exponential growth the protein number fractions, y_i, are constant in time one gets:

$$y_i = \frac{P_i}{\sum_k P_k} = \frac{u_i}{T_i^{\text{mod}}} / \sum_k \frac{u_k}{T_k^{\text{mod}}} \qquad \text{Eq. 56S}$$

It then follows from Eq. 56S that:

$$\frac{L_i P_i}{\sum_k P_k} = \frac{u_i / \tau_i}{\sum_k (u_k / T_k^{\text{mod}})} \qquad \text{Eq. 57S}$$

Summing up one gets:

$$\frac{\sum_i M_i}{\sum_i P_i} = \frac{\sum_i u_i / \tau_i}{\sum_i u_i / T_i^{\text{mod}}} \qquad \text{Eq. 58S}$$

Or:

$$\frac{1}{P_{tot}}\sum_i \frac{u_i}{T_i^{\mathrm{mod}}} = \frac{1}{M}\sum_i \frac{u_i}{\tau_i} \quad \text{Eq. 59S}$$

This proves the validity of Eq. 54S.

REFERENCES

1. O'Connor, P.B., Andreev, D.E. and Baranov, P.V. (2016) Comparative survey of the relative impact of mRNA features on local ribosome profiling read density. *Nat Commun*, **7**, 12915.
2. Levenberg, K. (1944) A Method for the Solution of Certain Non-Linear Problems in Least Squares. *Quarterly Appl Math*, **2**, 164-168.
3. Marquardt, D. (1963) An Algorithm for Least-Squares Estimation of Nonlinear Parameters. *SIAM J Appl Math*, **11**, 431-441.
4. Martin, D.C. and Katti, S.K. (1962) Approximations to the Neyman Type A Distribution for Practical Problems. *Biometrics*, **18**, 354-364.
5. Bremer, H. and Dennis, P.P. (2008) In Böck, A., Curtiss III, R., Kaper, J. B., Karp, P. D., Neidhardt, F. C., Nyström, T., Slauch, J. M., Squires, C. L., Ussery, D. and Schaechter, E. (eds.), *EcoSal-Escherichia coli and Salmonella: Cellular and Molecular Biology. http://www.ecosal.org*. ASM press, Washington, DC.
6. Pavlov, M.Y. and Ehrenberg, M. (2013) Optimal control of gene expression for fast proteome adaptation to environmental change. *Proc Natl Acad Sci USA*, **110**, 20527-20532.
